# Partial homologies between sleep states in lizards, mammals, and birds suggest a complex evolution of sleep states in amniotes

**DOI:** 10.1371/journal.pbio.2005982

**Published:** 2018-10-11

**Authors:** Paul-Antoine Libourel, Baptiste Barrillot, Sébastien Arthaud, Bertrand Massot, Anne-Laure Morel, Olivier Beuf, Anthony Herrel, Pierre-Hervé Luppi

**Affiliations:** 1 Neuroscience Research Center of Lyon, SLEEP Team, UMR 5292 CNRS/U1028 INSERM, Université Claude Bernard Lyon 1, Lyon, France; 2 Nanotechnologies Institute of Lyon, UMR5270 CNRS, INSA Lyon, Université Claude Bernard Lyon 1, France; 3 Health Image Processing and Acquisition Research Center of Lyon, UMR 5220 CNRS/U1206 INSERM, INSA Lyon, Université Claude Bernard Lyon 1, LYON, France; 4 MECADEV, UMR7179 CNRS, National Museum of Natural History, Paris, France; 5 University of Antwerp, Department of Biology, Antwerpen, Belgium; 6 Ghent University, Evolutionary Morphology of Vertebrates, Ghent, Belgium; University Tübingen, Germany

## Abstract

It is crucial to determine whether rapid eye movement (REM) sleep and slow-wave sleep (SWS) (or non-REM sleep), identified in most mammals and birds, also exist in lizards, as they share a common ancestor with these groups. Recently, a study in the bearded dragon (*P*. *vitticeps)* reported states analogous to REM and SWS alternating in a surprisingly regular 80-s period, suggesting a common origin of the two sleep states across amniotes. We first confirmed these results in the bearded dragon with deep brain recordings and electro-oculogram (EOG) recordings. Then, to confirm a common origin and more finely characterize sleep in lizards, we developed a multiparametric approach in the tegu lizard, a species never recorded to date. We recorded EOG, electromyogram (EMG), heart rate, and local field potentials (LFPs) and included data on arousal thresholds, sleep deprivation, and pharmacological treatments with fluoxetine, a serotonin reuptake blocker that suppresses REM sleep in mammals. As in the bearded dragon, we demonstrate the existence of two sleep states in tegu lizards. However, no clear periodicity is apparent. The first sleep state (S1 sleep) showed high-amplitude isolated sharp waves, and the second sleep state (S2 sleep) displayed 15-Hz oscillations, isolated ocular movements, and a decrease in heart rate variability and muscle tone compared to S1. Fluoxetine treatment induced a significant decrease in S2 quantities and in the number of sharp waves in S1. Because S2 sleep is characterized by the presence of ocular movements and is inhibited by a serotonin reuptake inhibitor, as is REM sleep in birds and mammals, it might be analogous to this state. However, S2 displays a type of oscillation never previously reported and does not display a desynchronized electroencephalogram (EEG) as is observed in the bearded dragons, mammals, and birds. This suggests that the phenotype of sleep states and possibly their role can differ even between closely related species. Finally, our results suggest a common origin of two sleep states in amniotes. Yet, they also highlight a diversity of sleep phenotypes across lizards, demonstrating that the evolution of sleep states is more complex than previously thought.

## Author summary

Until recently, the general understanding about sleep was that only mammals and birds show two sleep states: slow-wave sleep and rapid eye movement (REM) sleep. Consequently, it was thought that these two states appeared independently in these warm-blooded animals. However, a recent paper reported the presence of these two states in the bearded dragon lizard (*Pogona vitticeps*), suggesting that these two states arose with the common ancestor of mammals, birds, and reptiles. We confirmed the presence of two sleep states in the bearded dragon and compared its sleep with that of another lizard, the Argentine tegu (*Salvator merianae*). Our results show that both lizard species have two sleep states with similarities to the two sleep states observed in mammals and birds. Additionally, our study of behavioral and physiological parameters as well as the brain activity associated with sleep in these lizards allowed us to also show important differences between these two species of lizards and between lizards, birds, and mammals. Our findings indicate that sleep in lizards is more complex than previously thought and raise further questions about the nature, function, and evolution of these two sleep states.

## Introduction

### Behavioral sleep

Based on the 1913 behavioral definition [1], sleep is characterized by sustained immobility, a species-specific sleep posture and location, and a high arousal threshold. In addition, it displays a circadian distribution and is homeostatically regulated. Based on these criteria, it has been shown that sleep occurs in all animals, from the simplest organisms to the most complex ones [2–5]. Such ubiquity of sleep indicates that it constitutes a fundamental need for all living organisms.

### Two sleep states in mammals and birds

**Fig 1:**
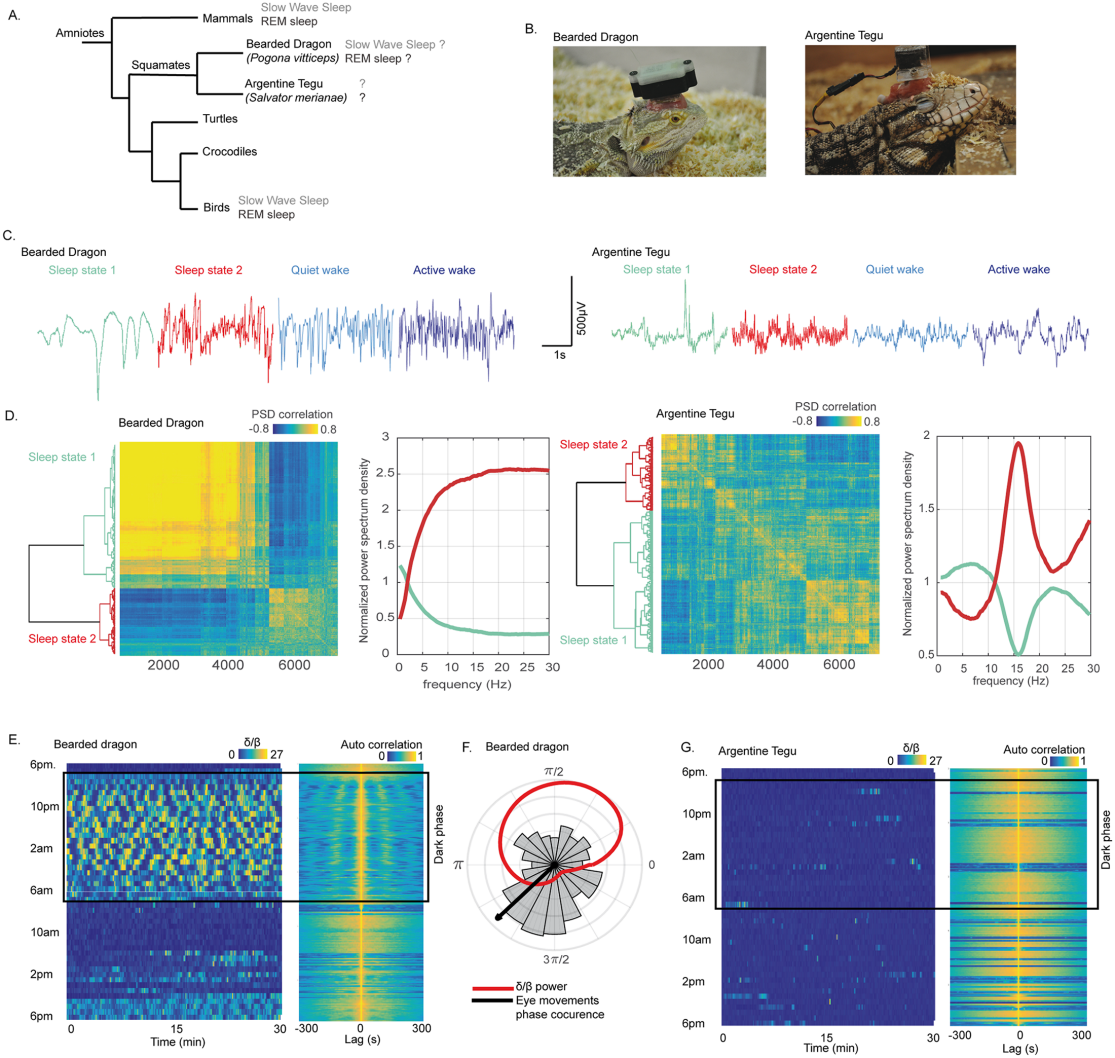
The tegu lizard does not sleep like a bearded dragon. (A) Phylogenetic tree of amniotes representing the common origin of mammals, birds, and nonavian reptiles. The figure also illustrates that both REM sleep and SWS have been identified in mammals and birds and more recently possibly also in a lizard, the bearded dragon (*P*. *vitticeps*). We investigated sleep in the Argentine tegu (*S*. *merianae*) to determine whether the bearded dragon is the only lizard to display those two sleep states. (B) Pictures of the two species recorded. The animals were equipped with a recording device on their head. (C) Raw signal of one LFP recorded in the DVR with a 35-μm–diameter tungsten electrode in the two species during S1 (sharing similarities with mammalian SWS) in bluish, S2 (sharing similarities with mammalian REM sleep) in red, QW in light blue, and AW in dark blue. The raw data illustrate the difference in all states of the DVR LFP. (D) Dendrogram (left) and correlation map (right) obtained from the hierarchical clustering of the distance between the correlation of each LFP 3-s window PSD between 9 PM and 2 AM in both species. On the right of each correlation map are the normalized mean power spectra of the two clusters computed for one animal in each species representing the two distinct sleep states identified, S1 in bluish and S2 in red. The comparison of the normalized power spectra of each state reveals a frequency profile that is clearly different between the two species, with a desynchronized activity (composed of all the frequencies higher than 5 Hz) for the bearded dragon during S2 and a power spectrum mainly composed of 15-Hz oscillations for the tegu. (E) The band power ratio (δ [0.5–4 Hz]/β [11–30 Hz]) computed as in Shein-Idelson and colleagues [22] for the bearded dragon. Each horizontal segment represents 30 min of the ratio computed with a 10-s window and a step of 0.1 s. The value of the ratio is color coded from 0 (blue) to 27 (yellow). The figure from the top to the bottom represents the evolution of the ratio over 24 h from 6 PM. A dark rectangle indicates the dark period. On the right, the normalized autocorrelation map of the ratio is illustrated. The autocorrelation was computed within 600-s windows with a step of 1 s. Both figures reveal a rhythmic alternance with a period of around 90 s across episodes, with δ frequencies (yellow) and episodes with β (blue) during the dark period, when the animal is lying on the floor with the eyes closed. (F) The distribution of the eye movements within each δ–β cycle; the mean phase is represented with a black arrow. The red line is the mean δ/β power ratio across the δ–β cycle. (G) This is the same figure as (E) for the Argentine tegu. The figure reveals no clear cycle in the δ/β power ratio over 24 h. AW, active wake; DVR, dorsoventricular ridge; LFP, local field potential; PSD, power spectrum density; QW, quiet wake; REM, rapid eye movement; SWS, slow-wave sleep; S1, sleep state 1; S2, sleep state 2.

In the 50s, two distinct sleep states were described in humans and cats [6,7]. The first sleep state is slow-wave sleep (SWS), also known as non-rapid eye movement (REM) sleep or quiet sleep. This state is characterized in mammals by the occurrence of cortical high-amplitude slow delta waves (0.5–4 Hz) [8], hippocampal sharp-wave ripple (hSWP-R) complexes [9,10], and spindle oscillations [11,12]. During SWS, physiological processes are reduced, including heart rate, body temperature, eye movements, and muscle tone. In sharp contrast, the active sleep state named REM or paradoxical sleep [7]—commonly associated with dreaming in humans—is characterized by REM and cortical desynchronization like the awake state, but without muscle tone [6,7]. REM sleep and SWS are also characterized by a high arousal threshold. During REM sleep, in contrast to SWS, thermoregulation processes including shivering, piloerection, and sweating are abolished [13], brain temperature increases, and the heart and breathing rates become irregular [14]. Finally, toe, tail, limb, and whisker movements occur phasically (muscle twitches) during REM sleep [15]. SWS and REM sleep have been unequivocally identified to date only in terrestrial mammals and birds [4] (Fig 1A). Since these species are homeotherms, it has often been proposed that the two sleep states evolved together with homeothermia [16]. However, the poikilothermic nonavian reptiles, including lizards and snakes, turtles, and crocodiles share a common ancestor with mammals and birds. Squamates (lizards and snakes) are the group that shares the most ancestral features with the common ancestor of birds and nonavian reptiles (Fig 1A). Therefore, to retrace the evolution of the two sleep states, studying species in this group is essential.

### Sleep in squamates (lizards and snakes)

Despite its importance in understanding the evolutionary origins of these sleep states, less than 40 studies, mostly from the 70s, have been devoted to the study of sleep in nonavian reptiles [4,17,18]. Of them, 16 were dedicated to squamates, with only seven articles including more than three recorded animals [19–25]. In addition, these studies were performed in only six species, all belonging to the infraorder Iguania. They revealed that this lizard family displays behavioral sleep during the night, including a specific posture and, when examined, a high arousal threshold and a homeostatic response to sleep deprivation. The sleep period was often described as one large bout during the night. During the day, periods of activity intersected long phases of quiet wake (QW). Sleep was also reported to be associated with a decrease of the heart and respiratory rates. Regarding the presence of one or multiple sleep states in lizards, the existence of a REM-like sleep state was already suggested in 1966 [19], mainly based on the presence of eye movements during sleep periods. However, these older studies failed to convince, and no consensus was obtained because of limitations in methodology, recording conditions, and the absence of replication [18]. However, in 2016, Shein-Idelson and colleagues provided convincing evidence for the existence of two electrophysiological sleep states [22] in a species never previously recorded for this purpose, the bearded dragon (*Pogona vitticeps*). The authors observed—specifically during the night, when the animal was lying on the floor of the cage with its eyes closed—a very regular alternation of periods characterized by the occurrence of “slow waves” and periods characterized by local field potential (LFP) desynchronization, similar to those observed during the awake state and associated with isolated eye movements. The authors concluded that both SWS and REM sleep exist in this species with a very rhythmic periodicity. However, such a periodicity, as regular as clockwork, is quite surprising and had never been reported before in either other nonavian reptiles or in mammals and birds. Moreover, muscle tone, motor automatism, heart rate, and arousal threshold evaluation were missing to unequivocally demonstrate that the state identified as REM sleep did not correspond to short periods of awakening also known to be characterized by desynchronized electroencephalogram (EEG) and eye movements.

Therefore, we decided to replicate the experiments of Shein-Idelson and colleagues [22] and to compare these data with data for another species of lizard from a different family to test the generality of these findings. We replicated data on one bearded dragon (*P*. *vitticeps*) (Fig 1B) and developed a multiparametric approach to examine sleep in the Argentine tegu lizard, *S*. *merianae* (Fig 1B). We chose this species as it belongs to the Lacertoidea family, for which sleep has never been recorded with the exception of three studies focusing on circadian rhythms [26–28]. Furthermore, this predatory species displays an active foraging life style, an omnivorous diet, and high cognitive abilities, with one of the highest encephalization quotients across squamates [29]. Consequently, it may have larger quantities of sleep and more specifically REM sleep than other lizards [30]. Six subadult Argentine tegus (*S*. *merianae*) were studied. We recorded LFPs by means of 35-μm–diameter tungsten electrodes implanted at different depths in four forebrain regions. We simultaneously recorded the nuchal electromyogram (EMG), the electro-oculogram (EOG), and the electrocardiogram (ECG) using a wireless system. All the animals were video monitored for 24 h a day with four near-infrared cameras. As brain LFP amplitudes and frequencies covary with temperature [31], we performed all the experiments at a constant temperature. Therefore, baseline conditions, the arousal threshold, and the effect of 9 h of sleep deprivation by means of gentle handling were recorded at 28 °C (body temperature). Finally, systemic injections of fluoxetine—a serotonin reuptake inhibitor known to suppress REM sleep in mammals [32,33]—were performed at two different concentrations.

## Results

### Replication of the bearded dragon sleep experiments

The signals obtained from tungsten electrodes implanted in the dorsoventricular ridge (DVR) of a bearded dragon—a forebrain structure proposed to be homologous to the mammalian isocortex, the amygdala, and/or the claustral complex [34–37]—revealed different patterns across vigilance states (Fig 1C). During the dark period, the bearded dragon displays a stereotypical posture, with the head lying on the floor in a specific location of the terrarium. This posture was never seen during the light period, as the animal always had its head up from the floor. During this period, two electrophysiological phases with distinct frequency content coexisted (Fig 1C and 1D). The first electrophysiological sleep state, rich in δ (0.5–4 Hz) frequencies, was characterized by a signal containing one to two slow negative high-amplitude sharp waves (HShWs) per second, lasting around 100–200 ms with an amplitude of 500 mV. The second electrophysiological sleep state contained frequencies in the β (11–30 Hz) band, an oscillatory pattern that looked like the awake one (Fig 1C). The δ/β power ratio and the autocorrelation of the signal revealed a very regular alternance between periods with δ and periods with β (Fig 1E). The periodicity of these cycles was around 90 s. Finally, the extraction of the occurrences of the eye movements from the EOG showed that the second electrophysiological sleep state contained more ocular movements than the first one. Eye movements were mainly isolated and appeared mostly at the beginning of S2 (Fig 1F). Our results obtained for one animal confirm the results reported by Shein-Idelson and colleagues. However, the same recordings and analysis performed on the Argentine tegu revealed different electrophysiological patterns (Fig 1C). Indeed, even if two electrophysiological sleep states could be detected during the night resting phase (Fig 1D), S1 did not contain slow negative HShWs as observed in the bearded dragon, and S2 differed from the awake activity, as an oscillation around 15 Hz dominates this phase. Finally, the autocorrelation analysis suggested no periodicity of the δ/β power ratio (Fig 1G). As the same protocol was performed on these two lizard species, and as it revealed such different results, we decided to characterize sleep in greater detail in the Argentine tegu and developed a multiparameter approach as described below.

### Behavioral sleep in the tegu is characterized by a decrease in the number of eye movements and a higher arousal threshold

**Fig 2:**
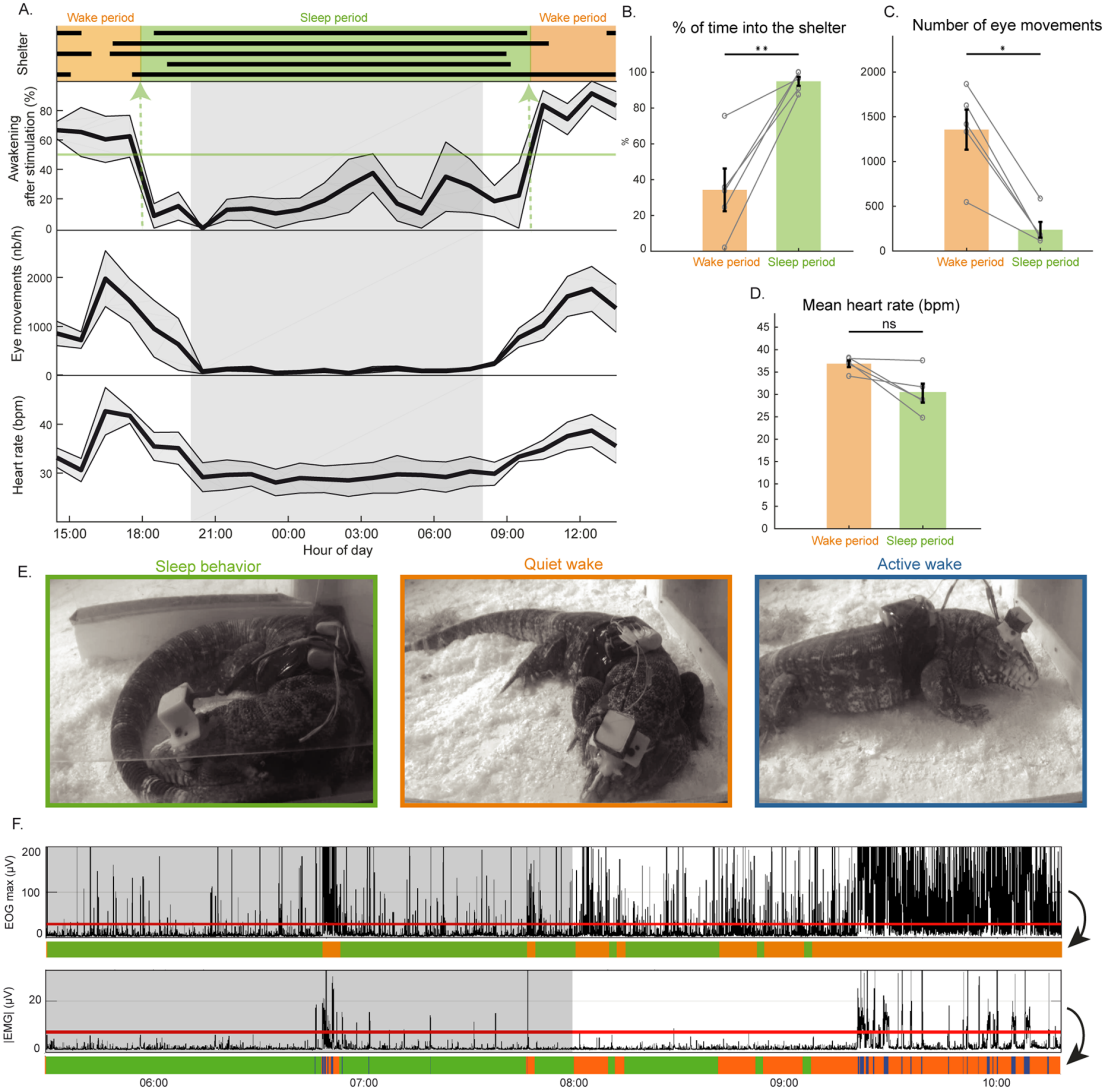
Behavioral sleep and automated scoring in the Argentine tegu (*S*. *merianae*). (A) The gray zone between 8 PM and 8 AM represents the dark phase. Representation over 24 h of the time spent inside (black bars) the shelter for each of the five animals recorded (top); mean ± SEM (line and gray zone) percentage of awakenings induced by a sensory stimulation made every hour (*n* = 5 animals); the mean ± SEM number of eye movements per hour; the mean ± SEM heart rate per hour. Choosing a threshold of 50% of awakening induced by the stimulation, we defined wake (in orange) and sleep periods (in green). (B) The sleep period significantly matched with the time passed inside the shelter (*p* = 0.0079). (C, D) A significant decrease of the number of eye movements per hour (*p* = 0.0159) and tendency of heart rate decrease (*p* = 0.0556) occurred during sleep compared to wake. (E) Positions and behavior of an animal during the three behavioral states identified: SB (green panel), with an animal in its shelter, the body curled up and eyes closed; QW (orange panel) with and animal outside the shelter lying on the floor, eyes often closed; and AW (blue panel) with an animal moving. (F) Graph illustrating the eye movements (maximal value of the EOG for each 1-s window during 5 h). The red line indicates the threshold used to differentiate SB (green) from wake (orange). The graph below represents the average of the absolute value of the EMG for the same period. The red line indicates the threshold used to differentiate QW (orange) and AW (blue). AW, active wake; EMG, electromyogram; EOG, electro-oculogram; QW, quiet wake; SB, sleep behavior.

During the light period, the tegus remained outside of their shelter and displayed short periods of active behavior, with head movements, locomotion, drinking, and feeding intersected by periods of immobility (quiet wake, QW) where animals were lying on the floor, eyes closed, head down with the four limbs spread apart. We observed that all animals entered their shelter 1 h (19 h 11 ± 27 min) before the onset of darkness (Fig 2A). Next, they curled up and kept their eyes closed and stayed in their shelter until 2 h after light onset (10 h 10 ± 23 min). During this phase, repositioning and movements of the head, limbs, toes, or whole body rarely occurred, and the eyes remained mostly closed. We also observed rare tongue flicking with the head slightly up and the eyes closed.

To objectively demonstrate that the animals were sleeping, we then measured for each hour the percentage of stimulations that induces an arousal and the associated number of eye movements and the heart rate. Between 6 PM and 10 AM, the percentage of time spent in the shelter was significantly higher than between 10 AM and 6 PM, while the number of stimuli that induced an awakening was significantly lower (*p* < 0.01). In addition, the number of eye movements and the heart rate tended to decrease during the night (*p* = 0.0556) (Fig 2A, 2C and 2D). In line with the behavioral definition of sleep, these results strongly suggest that the animals are awake between 10 AM and 6 PM and are sleeping between 6 PM and 10 AM. We then developed a custom script based on the number of eye movements (Fig 2F) and the muscle activity (S1 Fig) to automatically score sleep behavior (SB), QW, and active wake (AW) (Fig 2E). We compared the periods of time spent in the shelter with SB periods scored with our algorithm and obtained 87% of correct assignments, a sensitivity of 0.91, and a specificity of 0.87 (S1 Table). Using such automatic scoring, we measured the percentage of time spent in each state over 24 h: 6.4 ± 1% (AW), 29 ± 2% (QW), and 64.6 ± 2% (SB), with a mean bout duration of 0.5 ± 0.1 min (AW), 2.6 ± 0.2 min (QW), and 18.3 ± 1.6 min (SB).

### Multisite LFP recordings reveal the occurrence of slower frequencies during active wake compared to quiet wake and behavioral sleep

**Fig 3:**
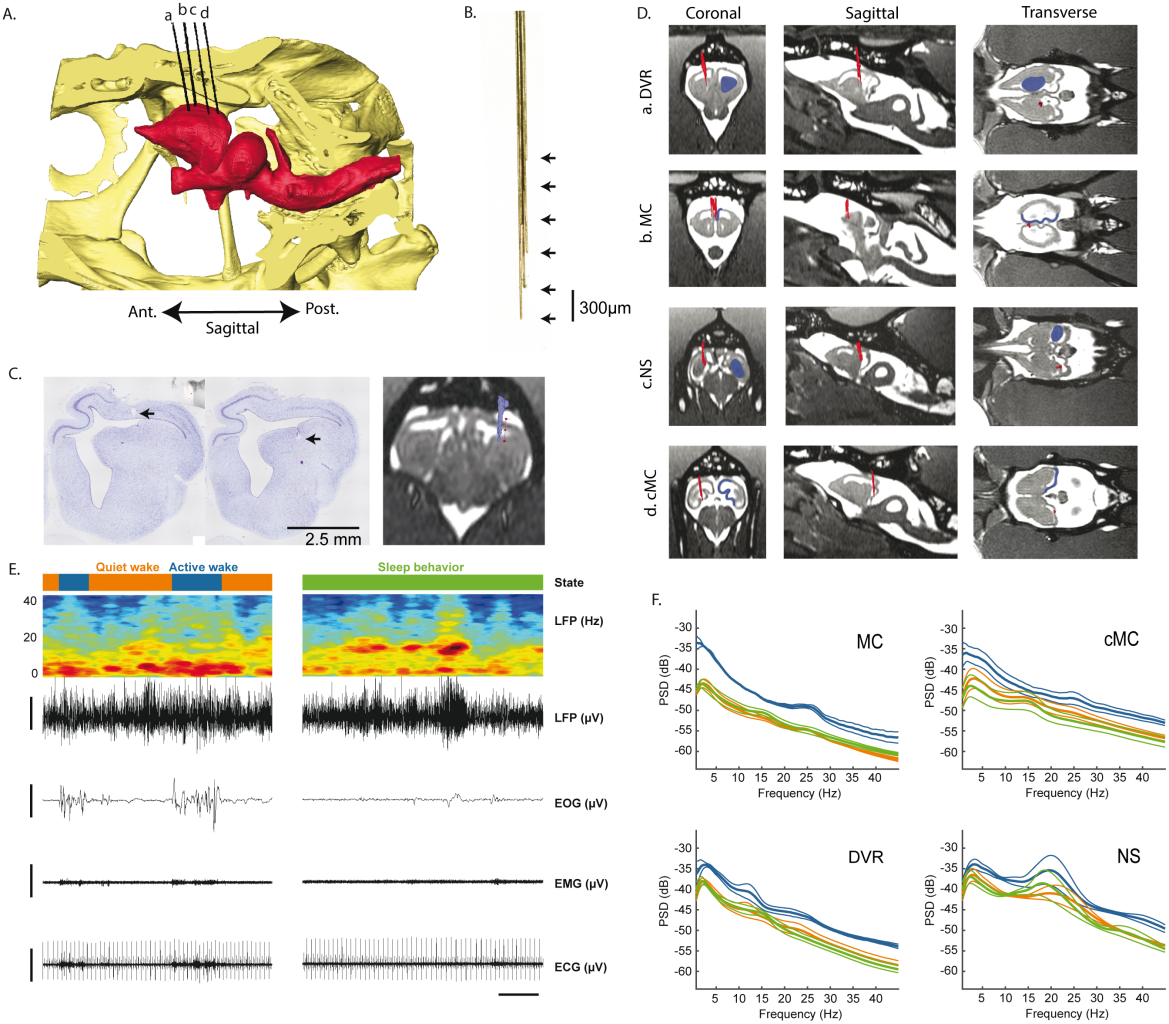
Electrode placement and raw signals. (A) 3D sagittal reconstruction of a lizard skull (in yellow) and brain (in red) based on in vivo presurgical MRI and CT scans. Electrode bundles (in black) were reconstructed from postsurgery CT scans. The electrodes a–d are, respectively, in the DVR, rMC, NS, and cMC. (B) Pictures of one of the bundles composed of six tungsten wires of 35-μm diameter spaced by 300 μm vertically. (C) Left and middle: 7-μm brain slices, labeled with a Nissl stain. The black arrow illustrates the lesions made by the electrodes implanted in the DVR. Right: corresponding electrode bundle reconstruction merged with the MRI. (D) Coronal, sagittal, and transverse MRI sections of each region implanted (in blue). The positions of the bundles of electrodes reconstructed from a CT scan and merged with the presurgical MRI are shown in red. (E) Left top to bottom: hypnogram of a period showing AW (in blue) and QW (in orange) states; the time frequency spectrogram representation of a DVR LFP recording (in blue the low power, in red the high power); the DVR LFP raw trace; the EOG filtered with a low-pass filter at 10 Hz; the EMG filtered with a high-pass filter at 10 Hz; the ECG filtered with a high-pass filter at 10 Hz. The horizontal scale bar represents 10 s, and the vertical scale bars 200 μV. It shows that AW is characterized by the predominance of low frequencies in the LFP, the presence of eye movements, and an increase in muscle activity and heart rate. During QW and SB states, the spectral composition is quite similar. (F) Mean ± SEM, power spectra across animals, computed for each state (blue, AW; orange, QW; green, SB) and each region. AW, active wake; cMC, caudal medial cortex; CT, computed tomography; DVR, dorsoventricular ridge; ECG, electrocardiogram; EMG, electromyogram; EOG, electro-oculogram; LFP, local field potential; NS, nucleus sphericus; QW, quiet wake; rMC, rostral medial cortex; SB, sleep behavior.

Baseline recordings of LFPs were made during 24 h at 28°C in the DVR, the rostral medial cortex (rMC) and caudal medial cortex (cMC)—homologous to the mammalian hippocampus [38]—and the nucleus sphericus (NS), a vomeronasal region [39] caudal to the DVR (Fig 3D and 3C). A 3D reconstruction of the coordinates of the brain structures and of the skull was made for each animal using in vivo MRI and computed tomography (CT) scans (Fig 3A) in order to accurately implant the targeted structures (Fig 3C and 3D). Bundles of 35-μm tungsten electrodes (Fig 3B) were implanted in these structures. The electrode positions were verified using a postimplantation CT scan merged with the preimplantation MRI and CT scans and postmortem histology (Fig 3C). The bundles consisted of four to eight electrodes covering 1,500 μm dorsoventrally (S2 Fig). In addition to the LFPs, we also recorded the EMG of the deep nuchal muscles, the EOG of both eyes, and the heart rate (Fig 3E).

During AW, a significantly higher muscle tone (*p* < 0.001), a higher number of eye movements (*p* < 0.001), and a higher heart rate (*p* < 0.001) were recorded compared to QW and SB (S1 Video). In addition, the LFP spectral power during AW was dominated by low frequencies (around 5 Hz) (Fig 3E and 3F). When comparing QW and SB, no significant difference was seen in muscle tone and heart rate variability. However, the heart rate significantly decreased during SB compared to QW (29.4 ± 2.6 versus 40.32 ± 1.2 bpm, *p* < 0.001). The LFPs in all regions showed a high diversity of patterns during all states and no obvious modifications of the mean power spectrum (Fig 3E and 3F; S1 and S2 Videos) except a small peak around 15 Hz during SB compared to QW in the DVR and rMC electrodes (Fig 3F). A large peak around 20 Hz was also clearly visible during all states, primarily in the NS (Fig 3F).

### Behavioral sleep in the tegu is composed of two states differentiated by sharp waves, 15-Hz oscillations, and eye movements

**Fig 4:**
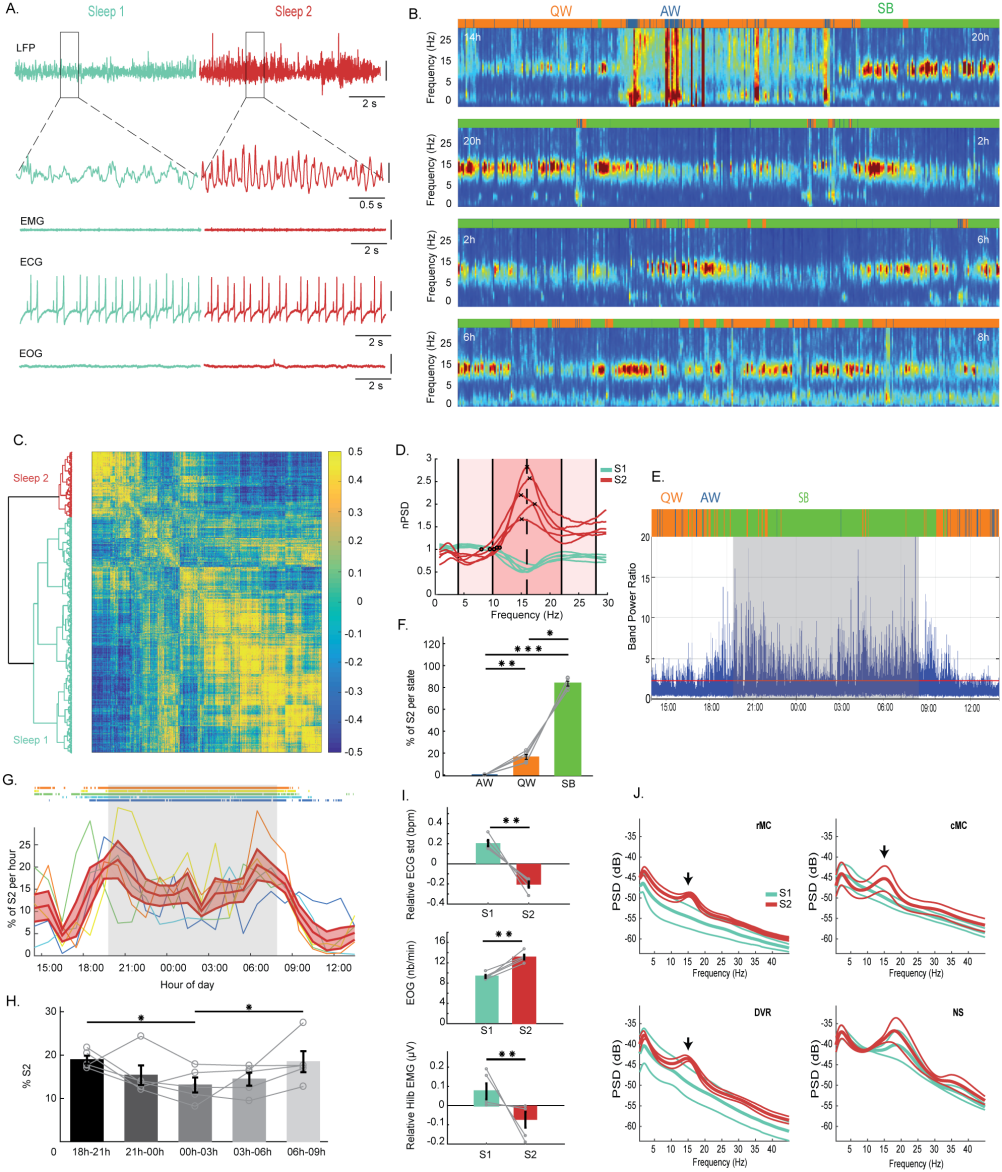
Two electrophysiological sleep states exist in the Argentine tegu. (A) From top to bottom: The first line shows raw LFP data obtained from an electrode located in the DVR during an S1 sleep period in bluish with no preeminent oscillations and an S2 sleep period in red showing 15-Hz oscillations. The lines below show an enlargement of the areas indicated by a square. The next lines show the corresponding muscle activity (EMG), heart rate (ECG), and EOG. The vertical scale bars represent 100 μV. (B) Time frequency representation of 24 h of recording of a whitened LFP DVR split into four 6-h periods starting at 2 PM (in blue the low power, in red the high power). The color bar above each time frequency illustrates the hypnogram obtained with automated scoring (blue for AW, orange for QW, and green for SB). The phasic occurrence, mainly during SB, of periods with oscillations around 15 Hz is clearly visible. In addition, the increase in low frequencies is clearly visible during AW. (C) Dendrogram (left) and correlation map (right) obtained from the hierarchical clustering of the distance between the correlation of an LFP 3-s–window power spectrum between 9 PM and 2 AM. (D) Mean power spectra of the two clusters computed for each animal, representing the two distinct sleep states identified, S1 in bluish and S2 in red. The black crosses are the frequency peaks of the mean power spectrum during S2, and the black circles the crossing between the power spectra of S1 and S2. The mean of these values is used to extract the S2 detection ratio, S2R = [10–22 Hz]/([4–10 Hz] + [22–28 Hz]). (E) Computation of the ratio S2R defined in (D) over 24 h in one animal. The red line is the threshold (the mean + 1 standard deviation of S2R) used to detect S2 state. (F) Percentage of S2 periods automatically detected during the three vigilance states, showing that S2 occurs mostly during SB (*n* = 5; SB versus QW, *p* = 0.0201; SB versus AW, *p* = 0.0002; AW versus QW, *p* = 0.0099). The gray lines represent the individual values for each animal. (G) Percentage of S2 periods per hour for each animal and the corresponding SB periods above. In red, mean percentage of S2 ± SEM across animals per hour during 24 h, showing that S2 periods tend to be more numerous at the beginning and at the end of the night than in the middle of it. (H) Histograms per 3 h showing that S2 periods occur significantly more at the beginning and at the end of the sleep time (from left to right, *p* = 0.0139, *p* = 0.0287, *n* = 5). (I) Histograms showing from top to bottom a significant decrease of the heart rate variability (*p* = 0.0079, *n* = 5); an increase in the number of eye movements (*p* = 0.0079, *n* = 5); and a small decrease in muscle tone computed as the norm of the Hilbert transform (*p* = 0.0079, *n* = 5) between S2 (red) and S1 (bluish) for each episode lasting more than two seconds. The heart rate variability and the muscle tone are represented relative to the mean muscle tone between S1 and S2 for each animal. The gray lines show individual values. (J) Mean ± SEM power spectra across animals for each region during S1 (bluish) and S2 (red), showing that the oscillation around 15 Hz characterizing S2 (arrows) is present in the DVR as well as in the rMC and cMC. In contrast, the 15-Hz oscillation is not visible in the NS, in which a 20-Hz oscillation occurs both during S1 and S2. AW, active wake; cMC, caudal medial cortex; DVR, dorsoventricular ridge; ECG, electrocardiogram; EMG, electromyogram; EOG, electro-oculogram; LFP, local field potential; NS, nucleus sphericus; QW, quiet wake; rMC, rostral medial cortex; SB, sleep behavior; S1, sleep state 1; S2, sleep state 2.

In agreement with the power spectrum analysis, we observed on the raw signal (Fig 4A) as well as on the time/frequency representation (Fig 4B, S2 Video) the phasic occurrence during SB of oscillations at a frequency of 15 Hz. We first selected for each animal the electrode showing the highest power of this 15-Hz frequency during SB using an unsupervised method (S3 and S4 Figs). We then performed a hierarchical clustering of the SB signals based on the correlations between each 3-s–window power spectrum for each animal. This revealed the existence of two clusters of sleep (Fig 4C). These two clusters define two electrophysiologically distinct sleep periods: S1 periods not showing any predominant oscillation and S2 periods characterized by the presence of an oscillation around 15 Hz (Fig 4D). Based on the mean power spectra of S1 and S2 computed for each animal, we extracted a power ratio (S2 detection ratio; S2R) to automatically detect the periods with 15-Hz oscillations (S2R = [10–22 Hz]/[(4–10 Hz) + (22–28 Hz)]) (Fig 4E). Periods displaying 15-Hz oscillations mostly occurred during sleep (83.4 ± 2%), although some were observed during QW (16.4 ± 2%). They were nearly absent during AW (0.09 ± 0.05%) (Fig 4F). The oscillations had a peak frequency of 15.3 ± 0.03 Hz and lasted on average 4.3 ± 0.1 s (but some episodes lasted 1 to 32 ± 2.3 s). They occurred 4.6 ± 0.1 times per min (2,229.6 ± 260 bouts over 24 h) without a regular periodicity, with an average individual variability of 3.4 oscillations per min ranging between 0.02 and 16. SB periods with these oscillations (S2) constituted 17.2 ± 2.3% of the total sleep time. No change in the power and the frequency of the oscillations was detected across the night. S2 periods occur preferentially at the beginning (18.9 ± 0.9%) and at the end (18.4 ± 2.4%) rather than in the middle (13 ± 1.7%, *p* = 0.0139 and *p* = 0.0287, respectively) of the night (Fig 4G and 4H). Further, S2 was associated with a lower heart rate (*p* = 0.0079, mean value: S1, 28.39 bpm; S2, 29.15 bpm; S1–S2, −0.24 bpm), lower heart rate variability (*p* = 0.0079, mean value: S1, 2.2 bpm; S2, 1.79 bpm; S1–S2, −0.41 bpm), a small but significant decrease in muscle tone (*p* = 0.0079, mean value: S1, 3.92 μV; S2, 3.77 μV; S1–S2, −0.15 μV), and an increase of the number of eye movements compared to S1 sleep periods (*p* = 0.0079, mean value: S1, 9.31 min^−1^; S2, 13.13 min^−1^; S1–S2, 3.82 min^−1^) (Fig 4I). Finally, the mean power spectra analysis revealed that the 15-Hz oscillations occurring during S2 were present in all regions except the NS (Fig 4J).

### HShWs occur specifically during S1 sleep periods

**Fig 5:**
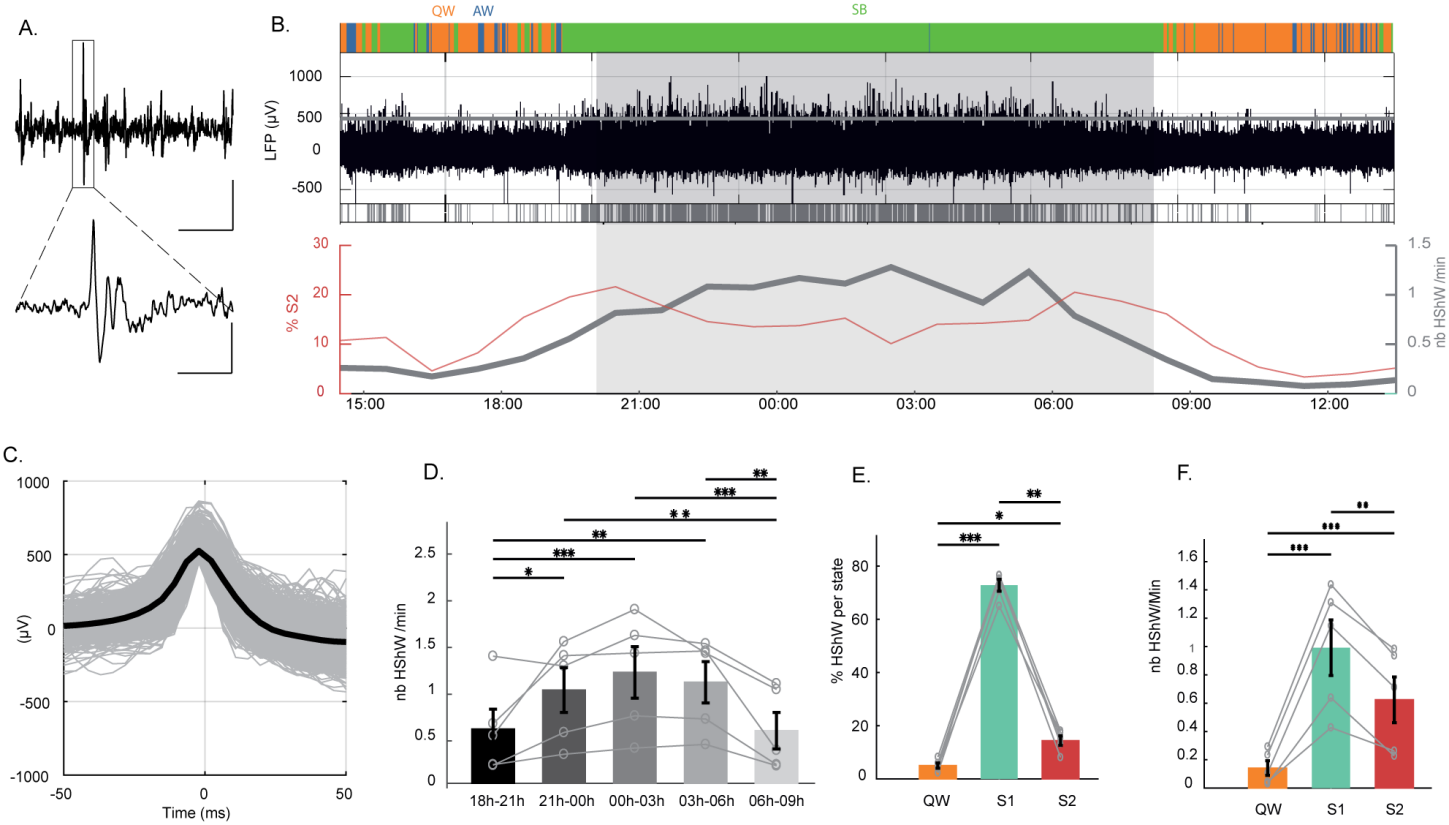
Sharp waves during sleep. (A) LFP raw trace showing an HShW at low (horizontal scale bar represents 5 s) and high magnification (horizontal scale bar represents 0.5 s in the square area shown at the bottom); the vertical scale bar represents 400 μV. (B) From top to bottom: hypnogram during 24 h, raw traces of a DVR LFP. The gray line corresponds to the threshold used to detect the HShWs. Below, a raster plot of the HShWs detected is shown in gray. The number of HShWs per min (in gray) and the percentage of S2 (in red) are shown. They appear to occur in antiphase. (C) All and mean waveforms of the HShWs detected by the automatic detection. (D) Histograms showing that HShWs occur more in the middle than at the beginning and at the end of the night (*n* = 5). From top to bottom: *p* = 0.0026, *p* = 0.0006, *p* = 0.0087, *p* = 0.0034, *p* = 0.0007, and *p* = 0.0112. The gray lines represent individual values. (E) Histograms showing that HShWs occur mainly during S1 (*n* = 5, QW [in orange] versus S1 [in bluish], *p* = 0.0002; QW versus S2 [in red], *p* = 0.0139; S1 versus S2, *p* = 0.0088). (F) Density of HShWs is also higher during S1 (*n* = 5; QW versus S1, *p* < 0.0001; QW versus S2, *p* = 0.0008; S1 versus S2, *p* = 0.0047). AW, active wake; DVR, dorsoventricular ridge; HShW, high-amplitude sharp wave; LFP, local field potential; QW, quiet wake; SB, sleep behavior; S1, sleep state 1; S2, sleep state 2.

HShWs were observed on LFPs from all structures (Fig 5A). They were extracted automatically from LFP signals using a spike-sorter algorithm [40] (Fig 5B and 5C). The HShWs displayed a mean amplitude of 635 ± 124 μV and lasted less than 50 ms (Fig 5C). They were significantly more numerous in the middle of the night between 0 AM and 3 AM (1.1 ± 0.2) than during the first (0.5 ± 0.1) and last 3 h of sleep (0.54 ± 0.1) (*p* < 0.001) (Fig 5B and 5D). They appeared mostly during S1 periods (72.8%), although some were visible during S2 (14.4%) and QW (5%) (Fig 5E and 5F).

### Sleep deprivation and an antidepressant suppressed HShWs and 15-Hz oscillations

**Fig 6:**
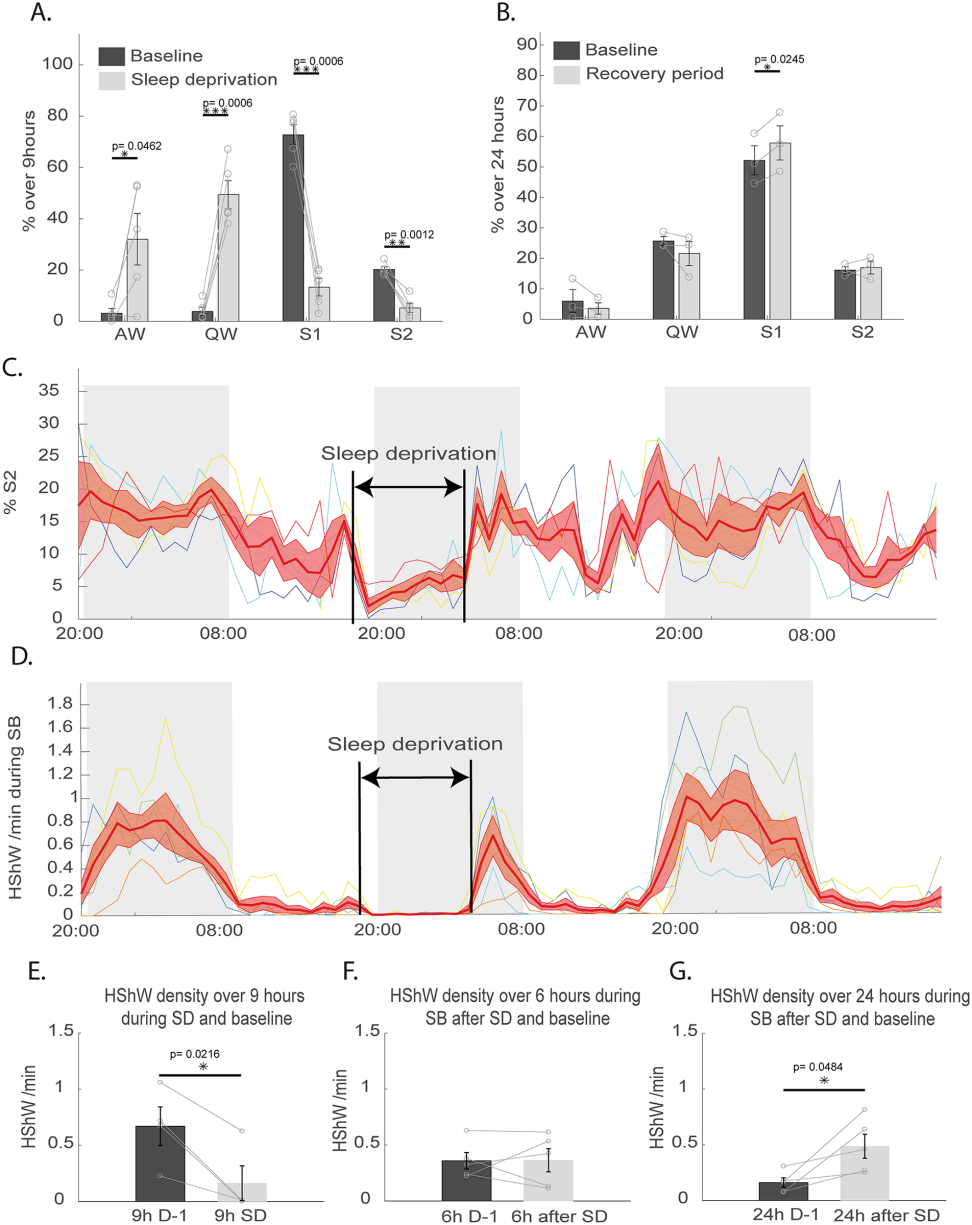
Effect of sleep deprivation on tegu sleep. (A) Quantification over 9 h of baseline (dark gray) and 9 h of sleep deprivation (light gray) of the percentage occupied by all states, including AW, QW, S1, and S2, showing the efficiency of SD. S1 was scored automatically based on the decrease of the eye movement density associated with a low muscle tone, and S2 was scored based on the 15-Hz oscillations. (B) Quantification over 24 h of baseline (dark gray) and the 24 h following the sleep deprivation (light gray) of the percentage occupied by all states, showing the recovery of S1. (C) Representation of the individual (thin colored lines) and mean ± SEM (large red line) percentage of S2 per hour, from the day before SD to the day after. The thin colored lines are the individual changes in such percentage. (D) Representation of the individual (thin colored lines) and means ± SEM (large red line) of the HShW density per hour, from the day before SD to the day after. (E) Histograms showing a significant reduction in the number of HShWs during sleep deprivation (*n* = 4, *p* = 0.0216). (F) No effect on the HShW density during the 6 h of SB following SD was detected, but a significant (G) increase was observed over 24 h after SD. AW, active wake; HShW, high-amplitude sharp wave; QW, quiet wake; SB, sleep behavior; SD, sleep deprivation; S1, sleep state 1; S2, sleep state 2.

To determine whether sleep homeostasis is present in lizards, 9 h gentle-handling sleep deprivation was performed between 7 PM and 4 AM (Fig 6A). During this sleep deprivation, the SB quantities (S1 + S2) were reduced significantly by 84.7 ± 4.8% compared to baseline conditions (Fig 6A and 6C) (*p* = 0.0006 for S1 and *p* = 0.0012 for S2). During sleep deprivation, the number of HShWs significantly decreased (Fig 6D and 6E) (*p* = 0.0216). After sleep deprivation, a significant increase of SB (Fig 6B; increase of SB: 8.96 ± 2.18%) occurred during the following 24 h compared to the baseline (*p* = 0.0302). The recovery of sleep was only significant for S1 (Fig 6B and 6C) (*p* = 0.0245). The density of HShWs during SB was significantly increased during the 24 h following the sleep deprivation compared to the baseline condition (Fig 6F and 6G).

**Fig 7:**
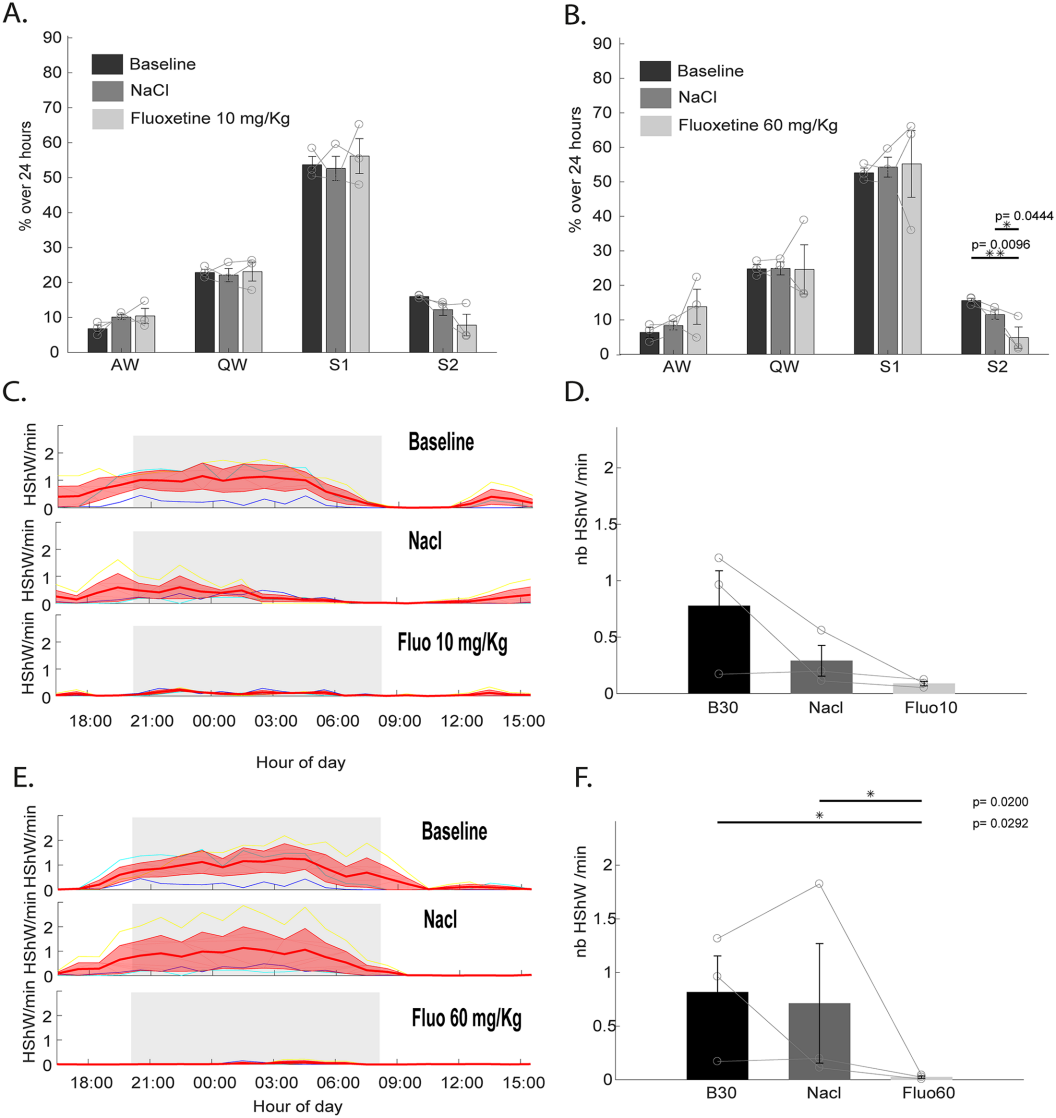
Effect of antidepressants on tegu sleep. (A) Quantification over 24 h of baseline (dark gray), following saline injection (medium gray), and following fluoxetine injection (10 mg/kg) (light gray) of the percentage occupied by all states, including AW, QW, S1, and S2. S1 was scored automatically based on the decrease of the eye movement density associated with a low muscle tone, and S2 scored based on the 15-Hz oscillations. (B) Quantification over 24 h of baseline (dark gray), following saline injection (medium gray), and following fluoxetine injection (60 mg/kg) (light gray) of the percentage occupied by all states. (C) Individual (thin colored lines) and mean ± SEM (large red line) changes of the HShW density per hour during 24 h of baseline, following saline injection, and following fluoxetine (10 mg/kg) injection. (D) Histograms showing that no significant change occurs between 6 PM and 10 AM in the number of HShWs after injection of NaCl or 10 mg/kg of fluoxetine at 5 PM. (E) Individual (thin colored lines) and mean ± SEM (large red line) changes of the HShW density per hour during 24 h of baseline, following saline injection, and following fluoxetine (60 mg/kg) injection. (F) Histograms showing a significant decrease of the number of HShW after injection of 60 mg/kg of fluoxetine compared to baseline and saline injections between 6 PM and 10 AM. AW, active wake; Fluo, fluoxetine; HShW, high-amplitude sharp wave; QW, quiet wake; S1, sleep state 1; S2, sleep state 2.

We then tested the effect of fluoxetine on the occurrence of the 15-Hz–oscillation periods defined as S2 and HShWs to determine whether they showed similarities with mammalian REM sleep and hippocampal sharp waves, respectively. Indeed, it has been shown that both REM sleep in mammals and birds [32,33,41] and in vitro hippocampal sharp waves are inhibited by serotonin reuptake inhibitors [42]. We injected fluoxetine [43,44] at two concentrations (10 mg/kg and 60 mg/kg) and a saline solution as a control (Fig 7). Control injection of saline did not induce any effect on the total percentage of SB, the number of SB episodes, or their duration compared to baseline (*p* > 0.05). The lower concentration of fluoxetine did not affect the total amount of AW, QW, and S1 (Fig 7A and 7B) (*p* > 0.05). Nevertheless, SB episodes (S1 + S2) were interrupted by short awakenings compared to baseline, inducing a significant decrease of their mean duration (10 mg/kg and 60 mg/kg, *p* = 0.0366 and *p* = 0.0255, respectively) and a significant increase in the number of SB episodes (10 mg/kg and 60 mg/kg, *p* = 0.0106 and *p* = 0.0325, respectively). Regarding the specific effect on the 15-Hz oscillations, their quantities were not significantly decreased with 10 mg/kg of fluoxetine in contrast to 60 mg/kg, strongly suggesting that the state of S2 is dramatically reduced (Fig 7A and 7B) (*p* = 0.0096). Regarding the effect on the HShW density (Fig 7C, 7D, 7E and 7F), both doses tended to reduce it during the 24 h after injection, but it was only significant for 60 mg/kg.

## Discussion

In the present paper, we confirm the findings of Shein-Idelson and colleagues on the bearded dragon [22]. Moreover, by coupling multisite LFPs, videos, and physiological recordings both under baseline condition and during and after sleep deprivation and fluoxetine injections, we were able to demonstrate the existence of two sleep states in the Argentine tegu. Further, we provide evidence of similarities between these two states in the tegu and mammalian and avian SWS and REM sleep. However, the phenotype of these two states in the tegu differs strongly to that observed in mammals, birds, and more surprisingly, the bearded dragon.

### Sleep in the bearded dragon

#### Behavioral sleep

Based on their posture and specific location, previous studies concluded that squamates display behavioral signs of sleep during the night. Yet, only four of these studies measured the arousal threshold [19–21,24], and only two used sleep deprivation experiments [20,24]. Even if arousal threshold and sleep deprivation were missing for the bearded dragon, the animal displayed a stereotypical posture with the head on the floor, the eyes closed, in a specific location of the terrarium. Thus, we suggest that in accordance with Shein-Idelson and colleagues, the animal was sleeping during this period.

#### Electrophysiological sleep

In this replication experiment, we confirmed that the bearded dragon displays two electrophysiological patterns corresponding to two sleep states. The first one, rich in δ frequencies, explained by the presence of slow HShWs, was proposed to be a homolog of the mammalian SWS. Indeed, this state is characterized by cortical delta waves and hippocampal sharp waves in mammals. However, whether those slow HShWs are similar to delta or sharp waves or those waves reflect a brain mechanism specific to the bearded dragon remains unknown. Complementary experiments, including cognitive tests and extracellular as well as intracellular recordings, should be conducted. Moreover, describing the memory network of nonavian reptiles—and specifically the implication of the DVR—is necessary to understand the role of these waves and to test whether they have the same cognitive role as in mammals. Regarding S2 as described by Shein-Idelson and colleagues, the DVR LFP looks like the awake state (Fig 1C), and isolated eye movements are more present during this phase. Based on these two features and the alternation with the SWS-like state, Shein-Idelson and colleagues proposed that this state is homologous to mammalian and avian REM sleep. If the animal is indeed sleeping, then this would be the most parsimonious hypothesis. However, eye movements and a desynchronized brain activity are also present during QW. An arousal threshold evaluation should be conducted to differentiate this putative REM-like sleep state from QW in this species. Nevertheless, the study by Shein-Idelson and colleagues provides credit to the hypothesis of the existence of an REM-like sleep state in squamates. The alternation between the two sleep states reported for the bearded dragon is also observed in mammals. However, a periodicity with a regularity like the one observed in the bearded dragon was never reported in either mammals or birds, which raises questions about its nature. Moreover, recent reports of artificial cyclical states with the same periodicity under urethane [45] or infra-slow–brain oscillations in the sigma band during mammalian SWS suggest that other ultradian cycles could exist in mammals [46] and possibly also squamates. Nevertheless, none of these cycles is associated with an increase in eye movements. Our replication confirms that the bearded dragon has two sleep states alternating with surprising regularity. However, at this point, we cannot draw conclusions about the nature and the homology of these states, and other species should be investigated to test the generality of these results.

### Sleep in the Argentine tegu

#### Behavioral sleep

Thanks to the evaluation of the behavioral criteria of sleep, we show for the first time that the Argentine tegu, the only species of the Lacertoidea studied so far, also displays behavioral sleep at night. As the animal spent (in these conditions) more than 90% of its time with its eyes closed and lying on the floor, it is difficult to differentiate SB from QW based on these features. We demonstrate here that SB differs from QW by a higher arousal threshold, a decrease in the number of eye movements, and a lower heart rate in addition to the specific location and posture typically observed. We also observed eye movements and occasionally small movements of the toes and the head. More often, large movements or repositioning of the animal during SB was also observed in the tegu, as reported for other lizards [18]. This would suggest that a state similar to REM sleep could be present in lizards. However, the nature of REM sleep could not be strictly identified from eye and other movements, as arousal also shows these features [47].

#### Does S1 sleep correspond to SWS?

Using our unsupervised, integrative, and multiparameter approach, we were able to distinguish two sleep states in the tegu during SB. S1 was characterized electrophysiologically by the absence of 15-Hz oscillations during behavioral sleep. In addition, numerous isolated HShWs occur at a rate of 1 per min in all structures recorded. The presence in the EEG of isolated sharp waves specifically during sleep has been already reported in other lizards [20,24,25]. Isolated sharp waves have also been recorded during sleep in turtles and crocodiles (for review, see [18]). Because of their morphology and their presence during sleep, it has been suggested that those waves could be similar to the mammalian hSWP-Rs (duration between 40–100 ms, amplitude that can exceed 2.5 mV, and a variable occurrence of 1 to 60 per min [10]). hSWP-Rs are generated by a burst of activity in cornu ammonis area 3 (CA3), inducing a large depolarization in cornu ammonis area 1 (CA1) stratum radiatum associated with a fast oscillation in the CA1 pyramidal layer [10]. Their role in the mammalian memory consolidation processes is well described [9,10]. Therefore, their existence in a reptilian brain during sleep would have important consequences regarding the function of sleep in these animals. However, the shape of the HShWs is also consistent with cortical slow waves. In the tegu, as well as in the bearded dragon, HShWs were reported during SB, just like hippocampal sharp waves and cortical slow waves in mammals. Regarding their morphology, the tegu and turtle HShWs show a similar duration: shorter than 50 ms, with an amplitude between 0.2 and 1 mV and an occurrence of 1 per min, similar to the mammalian hippocampal sharp waves. Interestingly, in contrast to the data reported in other lizards and nonavian reptiles, the bearded dragon showed slow HShWs occurring at a high rate of 60–120 per min (0.5–1 Hz) with a half width of 100–400 ms, morphologically rather similar to mammalian slow waves. Moreover, the localization of the tegu HShWs also questions their true nature. Indeed, mammalian hippocampal sharp waves are recorded in the mammalian hippocampus, subiculum, and entorhinal cortex [10] and were not reported in birds (for review, see [48]). In contrast, slow waves have been recorded in most cortical regions in mammals [49,50] and birds, as well as in the avian DVR during anesthesia [51]. As HShWs were observed in all recorded regions in the tegu forebrain, this could suggest that reptilian HShWs could be a precursor form of avian and mammalian slow waves. This hypothesis is also supported by a recent report of HShWs in the crocodilian DVR under anesthesia [52]. In reptiles, pharmacological experiments have also been conducted. In the 70s, injections of atropine sulfate, amphetamine, nembutal, alpha-methyl-tyrosine, and parachlorophenylalanine, drugs known to modify the quantity of ventral hippocampal sharp waves in the cat, were shown to induce the same effects on turtle sharp waves (*Geochelone carbonaria*) [53,54]. This let the authors suggest that reptilian HShWs could be similar to mammalian sharp waves. However, some of the drugs that suppress mammalian hippocampal sharp waves also suppress cortical slow waves. In the Argentine tegu, we demonstrated that the HShWs tend to disappear after fluoxetine injection. However, in mammals, little is known of the effect of serotonin on hippocampal ShWs. To our knowledge, only one paper reported that serotonin blocks, in vitro, rodent hippocampal sharp waves [42].

As a conclusion, since the precise mechanism of the generation of HShWs has not been identified, their definition relies on a rather vague description based on their shape, state of occurrence, occurrence rate, and pharmacological responsiveness. Unfortunately, these properties do not allow us to differentiate between hSWP-Rs and slow waves or another type of wave specific to lizards. However, as both hippocampal sharp waves and cortical slow waves are present during SWS in mammals, this would suggest that S1 in the tegu is likely homologous to mammalian and avian SWS. Moreover, after fluoxetine injection, the HShWs disappear (Fig 7), but the automated sleep-scoring algorithm (Fig 7A and 7B) suggests that the animal is able to sleep without these waves. This is further suggested by the typical sleep posture taken up by the animal inside its shelter. This suggests that the HShWs are only one feature of the complex phenotype of sleep in reptiles. Moreover, these waves by themselves are not sufficient to characterize this sleep state in reptiles.

#### Does S2 sleep correspond to REM sleep?

Based on the presence of active periods during sleep, the existence of REM sleep has been suggested in six of the seven previous studies on lizards. They mostly observed limb and eye movements associated with an EEG with an awake-like activity. In the tegu, we reported a state (S2) that shares partial similarities with mammalian and avian REM sleep. It is electrophysiologically characterized by the presence of oscillations with a 15-Hz frequency present in nearly all structures recorded during SB. To our knowledge, such a type of oscillation has never been reported before during sleep in any lizard species. We demonstrated that S2 episodes preferentially appear at the beginning and at the end of the sleep period and lasted 4.3 s on average. The 2,229.6 ± 261 episodes constituted 17.2 ± 2.3% of the total sleep time. Because some S2 episodes lasted more than 20 s, it is unlikely that the oscillations correspond to sleep spindles, an oscillation appearing during SWS in mammals in the same range of frequencies (between 10 and 18 Hz) and lasting between 0.4 and 1 s [11,12]. In addition, we observed that a higher density of eye movements and a decrease in muscle tone occurred during S2 compared to S1. We also showed that the 15-Hz oscillations, but not SB, were suppressed after the injection of fluoxetine, a specific serotonin reuptake inhibitor (Fig 7). This suggests that S2 was suppressed after 60 mg/kg of fluoxetine injection, although we cannot exclude that the drugs may have suppressed the oscillations but not the state. Contrary to such a hypothesis, we were not able to identify periods after fluoxetine injection displaying an increase of eye movements and a decrease of muscle tone as seen during S2. In addition, no significant changes were observed when comparing the muscle tone and the eye movement density during SB (S1 + S2) in the different conditions (EOG density during baseline versus saline versus fluoxetine 60 mg/kg, *p* = 0.6115; muscle tone, *p* = 0.416). Yet, even if the serotonin distribution in the brainstem is similar in lizards compared to mammals [55], and despite the fact that some studies on lizards have suggested a similar effect of fluoxetine on aggression [56], it remains unknown whether the S2 state shares the same neuronal substrate with mammalian REM sleep. Importantly, the duration and regular occurrence of REM sleep reported in the bearded dragon does not match that seen for S2 in the tegu nor that previously reported in birds and mammals. Indeed, in the tegu, the duration and temporal distribution of S2 are quite similar to that seen for REM sleep in most birds, which display a short mean duration of episodes of 5 to 10 s and a percentage of around 10% of total sleep time [57]. Another important feature of bird and mammalian REM sleep is the presence of ocular saccades. As reported here in the tegu from EOG recordings and in the bearded dragon based on unilateral video monitoring, more eye movements also occur specifically during the postulated REM sleep episodes. However, in both cases, the eye movements were isolated [22] and often unilateral in the tegu, in contrast to the REMs recorded in mammals that occur in bursts [58], illustrating that the phenotype of S2 is different from that of REM sleep in mammals and birds.

Altogether, our results for the tegu and those obtained for the bearded dragon suggest that two different sleep states with partial similarities to REM sleep and SWS exist in two different species of lizard, even if an arousal threshold evaluation would be necessary in both species to clearly differentiate S2 from QW. Yet, the short duration of the REM-like sleep state in the tegu renders this extremely difficult from a practical point of view. However, these two states display very different temporal distribution and type of oscillations in the two species. It therefore raises the question whether one or the other is the exception among lizards. The recording of additional lizard species is required to answer this question. Further, additional experiments are necessary to determine whether the structures generating REM-like sleep episodes in the bearded dragon and the tegu are the same as those generating REM sleep in birds and mammals. Moreover, the constant temperature used does not reflect the natural temperature fluctuations experienced by these animals and therefore constitutes a limitation of this study. Finally, we observed rare small and isolated twitches or motor automatisms in the tegu during the night but without a strict association with the S2 state. It might be that these muscle twitches occur during short periods of awakening or that in tegu, twitches are not associated to a specific sleep state. Another possibility is that they occur specifically during S2 only in young animals, as it has been shown that they are more numerous during this stage in mammals [59,60]. In agreement with this hypothesis, more muscle twitches have been observed in juvenile lizards and even in ovo [61]. To summarize, the two species of lizards recorded displayed two sleep states sharing some similarity with mammalian and avian REM sleep and SWS but diverged notably regarding the presence of twitches, the speed and number of the eye movements, and the absence of a wake-like EEG for the tegu compared to mammalian REM sleep.

### Implications for the origin of the sleep states

Our results demonstrate the existence of two different sleep states in the tegu and the bearded dragon, sharing features with mammalian and bird REM sleep and SWS. The existence of an REM-like sleep state in a lizard suggests that homeothermic animals are not the only ones to show two sleep states. However, even if some nonavian reptiles display two sleep states, the ancestral or convergent origin of these states remains unclear. In fact, too few studies have been conducted in nonavian reptiles to fully conclude that a REM-like sleep state did not appear convergently. Moreover, around 75% of the studies on turtles and almost all of the studies on crocodiles (both groups being closely related to birds) did not report two sleep states. Whether the two sleep states originated at the base of the amniote tree or before also remains to be determined by means of new studies of sleep in other nonavian reptiles as well as amphibians and fish. Deciphering the origin of the two sleep states is complicated, and the further we move away from mammals and the classical definition of sleep, the more difficult it will be to identify homologies. More than providing additional evidence for a reptilian REM-like sleep state, our results reveal the true diversity in sleep phenotypes, a diversity that should be explored through integrated and complementary approaches without an underlying biased definition based on mammalian studies. Indeed, even in mammals and birds, experiments on basal species show that those states could be mixed [62,63]. Maybe the question should not be whether nonavian reptiles show REM sleep and SWS, but how did these states appear and evolve along the different branches of the amniote tree.

## Materials and methods

### Ethics statement

All experiments were conducted according to the 3R principles in animal experimentation and in accordance with the European Community Council Directive for the use of research animals (2010/63/EEC; https://eur-lex.europa.eu/legal-content/EN/TXT/?uri=CELEX:32010L0063). Protocols and procedures used were approved by the local ethics committee for animal experimentation of the university Lyon 1 (No. BH2012-43).

### Animals

We report data on one bearded dragon (*P*. *vitticeps*) and six Argentine tegus (*S*. *merianae*), five males and one female (#2), with an age of 2 y (± 0.5), 3 ± 0.7 kg. All tegus were bought from official breeders and were maintained individually in a 4-m-square area (2 m × 2 m). The bearded dragon was maintained and recorded in a smaller terrarium (90 cm length, 50 cm width, 40 cm height). The tegus were fed dead mice two to three times a week, and the bearded dragon was fed crickets and vegetables twice weekly. Water was provided ad libitum. Prior to experiments, animals were maintained under a 12 h:12 h light/dark cycle in a room maintained at 25 °C with a hot spot at 45 °C available between 11 AM and 6 PM. Six infrared (850 nm) panels (Viewpoint SA) were always on. A shelter transparent to infrared wavelengths was used to monitor the animals during the dark phase. All the experiments were conducted in a room at 25 °C after at least 2 days of habituation. A custom floor heating regulated at 30 °C was used for the tegu. The nuchal temperature was measured for one animal of each species thanks to a micro thermistor implanted in the nuchal muscles. The nuchal temperature measured in one animal was around 28 °C for the tegu and 25 °C for the bearded dragon.

### Imaging and verification of the electrode position

Prior to the surgery, two 100-μm diameter holes were drilled under anesthesia (cf. surgery part) in the anterior and posterior part of the parietal bone. These holes served as references during electrode implantation. MRI imaging was carried out on a 3T GEHC MR750 System using an 8-channel wrist coil. The head of the lizard was placed at the center of the coil. After a three-plane localizer, two 3D high-spatial–resolution magnetic resonance imaging (HR-MRI) acquisition sequences were performed in the coronal plane. For both HR-MRI acquisitions, similar parameters were used: 59.2-mm slab thickness with 100 × 80 mm^2^ field of view (FOV), 148 × 448 × 384 acquisition matrix size, and a 592 × 1,024 × 768 reconstruction matrix leading to a slice thickness of 100 μm with an in-plane pixel of 97 × 97 μm^2^. First, a T1-weighted FSGGR sequence with 30 ° flip angle, 29.4 ms TR, 10.1 ms TE, and ±7.8k Hz receiver bandwidth with 22’15” scan time was performed. Second, a FIESTA-C sequence with 70 ° flip angle, 10.8 ms TR, 3.6 ms TE, and ±41.7 kHz receiver bandwidth with 12’45” scan time was performed. A few days later, a CT scan was performed to image the skull. The experiments were done on an NVEON system (Siemens), with a tension of 80 kV, a current of 500 μA, and an exposure time of 900 ms with 720 steps. The reconstruction of the final volume permits us to obtain a voxel size of 55.62 μm^3^ in the three dimensions. The two modalities (MRI and CT scan) were realigned by choosing at least 10 common landmarks and using a principal component analysis method for realignment (Avizo v7.0.1). Next, landmarks were put on the MRI slices at the targeted electrode positions. A custom script (Matlab r2016b; The MathWorks, Natick, MA, USA) was used to transform the targeted landmarks into the reference frame defined by the holes drilled on the skull. The coordinates obtained were those used for the surgery. This procedure was used for all animals of both species. One week after the surgery, a second CT scan was performed in order to check the electrode positions by realigning the last CT scan to the two presurgical images.

### Perfusion

Under surgical anesthesia (cf. surgery) and after an electrocoagulation lesioning (2 s, 0.5 mA), animals were perfused transcardially [64] with a 400-ml Ringer-Lactate solution (Braun Medical, France) followed by a 2,000-ml 4% paraformaldehyde fixative solution, with a perfusion pump (Gilson, France) set at a 55 ml/min rate. Brains were removed and postfixed for 2 d at 4 °C in the same solution. Brains were included in paraffin (LEICA ASP300, Germany) and mounted (Myr EC 350–2, Spain), and 7-μm slices were cut with a microtome (Leica RM2245, Germany) for histochemical processing. One slice out of every seven was kept.

### Nissl staining

The Nissl staining was done for the bearded dragon and tegus #2, #3, #4, #5, and #6. The paraffin was removed from each slice with two 4-min baths of methylcylohexane, then two 4-min baths of 100% alcohol, followed by a 4-min bath of water. The Nissl stain was performed by putting the slices successively into the following baths: 2 min water, 4 min Cresyl Violet acetate (1 g/L) (Sigma Aldrich, St. Louis, MO, USA), 1 min alcohol 75%, 30 s alcohol 95%, 15 min alcohol 15%, 5 s alcohol 100%, 5 s alcohol 100%, 2 min OTTIX (MM France, France). Then slices were digitalized (Zeiss Axioscan Z1, Germany) with a 5X Fluar (ON 0.25) lens.

### Surgery

The animals were anesthetized with a mixture of ketamine (66 to 100 mg/kg) and medetomidine (100 to 200 μg/kg) at 19 °C injected intramuscularly and equally distributed in the four limbs [65]. After every 6 h, reinjections of half the previous dose were performed. Reflexes and respiratory rate were checked throughout the surgery. During surgery, two stainless steel electrodes were inserted bilaterally in the intercostal muscles for measuring heart rate, and two others were also implanted in the neck muscles to assess muscle tone. For the tegus and the bearded dragon, two other electrodes, gold plated at the tip, were positioned behind each eye, under the eyelid, to record eye movements. The tegus were also implanted with three to four bundles of six 35-μm diameter tungsten electrodes in different brain regions (DVR, NS, rMC, and/or cMC). Only the DVR was implanted with this kind of bundle during the bearded dragon surgery. One screw was fixed on the skull between the two eyes for signal referencing for the tegu. The reference was inserted on the most caudal part of the parietal bone for the bearded dragon. To do so, lizards were placed in an adapted stereotactic frame. All wires were then connected to a head connector (EIB-36-PTB Neuralynx), which was secured over the skull using acrylic Superbond (Sun Medical Co.). Next, dental Paladur cement (Heraeus Kuzler) was applied around the head connector to protect all the wires and the connector.

### Behavior

The behavior of the animal was monitored with four cameras (Dragonfly2 DR2-HIBW, PointGrey) equipped with a band-pass filter in the near-infrared wavelength. One camera was recording the full area, and the three others were dedicated to the animal shelter. The videos were recorded 24 h a day (VPCore2, Viewpoint), and the actimetry, which is the number of pixels changing more than 14 gray levels between two successive images, was evaluated online for each camera.

### Arousal threshold

The arousal threshold was evaluated for the Argentine tegus. A microrotor was fixed over the head of the animal for at least 4 d. The microrotor was programmed with a custom device to rotate at the maximum power for 5 s every hour to avoid any habituation. When the rotor was activated, an LED light was on. Using the four videos, any signs of awakening (like an eye opening or a leg or head movement) after a stimulation was recorded, as well as the latency thereof. The percentage of awakening after stimulation was evaluated for each hour for each animal. The mean percentage was then calculated for five animals (Fig 2A).

### In vivo electrophysiology

For the tegus, the electrophysiological signals from at least 22 tungsten electrodes into the brain—two ECG, two EMG, two EOG, and in some animals, a screw EEG—were recorded wirelessly (TBSI W32). A custom battery (3,000 mAh) for recording at least 4 d without changing the battery was used and fixed over the back of the animal with tape. The amplification of the system was 1,000×. The digitalization was performed using a DAQ card (National Instruments USB 6363) with a custom script (Matlab r2016b). The data were sampled first at 20 kHz, low-pass filtered at 500 Hz, and subsampled at 2 kHz online. The videos were synchronized with an output TTL to trig the start and the stop of each video. For the bearded dragon, the DVR LFP was recorded with eight tungsten electrodes at different depth (−4 to −2 mm below the skull, at 1.28 mm caudal to the anterior part of the pineal hole, and 1.94 mm lateral). Two EOGs were also recorded. The signals were recorded thanks to a custom wireless recording device at 128 Hz and to a custom Matlab script.

### Sleep deprivation

The sleep deprivation was performed on the Argentine tegu by gentle handling without changing the light cycle from 7 PM to 4 AM. The shelter was removed from the area, and when the animal displayed a sign of sleep (mostly closing the eyes), the experimenter woke up the animal by pulling a rope attached to the animal’s tail. After deprivation, the animal was left for at least 24 h without any human intervention. The sleep deprivation was performed on four animals, and the recordings started during the baseline and ended at least 24 h after the recovery.

### Pharmacology

Twelve ml of fluoxetine (10 and 60 mg/kg; Interchim, France) or saline (vehicle) solutions were randomly injected intraperitoneally at 4 PM in the tegus. Animals were recorded for at least 48 h after injections. Each injection was spaced at least 2 d apart for NaCl and 3 d after the fluoxetine injections.

### Preprocessing, visualization, and “shelter scoring”

All the electrophysiological signals of the tegus were filtered with a zero phase-shift low-pass filter (cutoff frequency 100 Hz, order 2) and subsampled at 250 Hz before any other treatments. Next, the electrophysiological signals, the actimetry, and the video were imported into a custom software program (SlipAnalysis, developed under Matlab r2016b). An empty hypnogram was then created. The hypnogram was then manually filled per 5 s from the video with two states: animal inside the shelter or animal outside the shelter. All the analyses performed were also done with custom scripts (Matlab r2016b).

### Automated vigilance states scoring

For the Argentine tegus, differential EOG calculated from the subtraction between the two EOGs was filtered with a low-pass filter (Fc 10 Hz, order 10). Then, the maximal value of the redressed signal was evaluated every second. Eye movement occurrence and duration were extracted by taking any part of the signal higher than 30 μV. Next, every epoch of the hypnogram during which the interval between eye movements was higher than 30 s was scored as SB. The other epochs were considered as QW. The episodes of SB spaced by less than 2 min were merged, and those lasting less than 2 min were removed. A differential EMG calculated from the subtraction between two EMGs was filtered with a high-pass filter (Fc 10 Hz, order 10) and the absolute value of the Hilbert transform was calculated. An average filter with a 0.5-s window was applied, and the mean value was evaluated for every 1 s bout. AW was scored when the processed EMG value was above 20 μV. Every episode lasting less than 5 s was ignored, and episodes spaced by less than 5 s were merged (Fig 2D and S1 Fig). For the baseline experiments, the episodes scored as SB in the automated hypnograms were compared with the “shelter scoring” (S1 Table). A mean correct rate of 0.873 was obtained on the six animals, with a mean sensitivity of 0.911 and a mean specificity of 0.874.

### Electrode selection

At least 22 electrodes were implanted in three to four regions in each tegu. In order to remove electrodes that were likely in the cerebral spinal fluid (CSF), we computed the mean power spectrum density (MPSD) into the 0.5–45 Hz band. For all animals, based on the imaging (MRI and postsurgery CT scan), we labeled the electrodes that were in the CSF and those in the brain. A threshold was obtained by computing the mean plus one standard deviation from all MPSD of the CSF electrodes. Every electrode with MPSD below the threshold was removed from the analyses and considered as being in the CSF (S2 Fig). In order to choose the best electrode to extract the S2 states, we computed the MPSD during the episodes scored as SB. An interpolated spectrum was computed by removing the 10–20 Hz band and keeping the value in the 5–10 Hz and 20–25 Hz bands (S3A–S3F Fig). A spline interpolation was used to evaluate this interpolated spectrum. By this means, one electrode was chosen per animal by taking the electrode with the maximal ratio between the interpolated and the real spectrum into the 10–20 Hz band. Animal two was removed from the S2 and HShW analysis because none of its electrodes had a ratio higher than 0.5%. As only the DVR was recorded, we choose the electrode with the highest amplitude for the bearded dragon.

### Clustering and S2 extraction

We used the methodology of Shein-Idelson and colleagues [22]. From the baseline experiments, between 9 PM and 2 AM, the signal of the chosen electrode was whitened with an autoregressive algorithm. A multitaper power spectrum between 0.5 and 30 Hz was computed for each 3-s epoch scored as SB (windows 3 s, bandwidth 1 Hz, 5 tapers [66]). Each power spectrum was normalized by the mean power spectrum. A correlation matrix of these power spectra was calculated. Then, a hierarchical clustering with two clusters was realized based on a Euclidian distance of the correlation and using a Ward linkage (Figs 1D and 4C). A mean normalized power spectrum per animal was then calculated for each cluster (Figs 1D and 4D). For Fig 1, we used the ratio used by Shein-Idelson and colleagues (δ/β, [0.5–4 Hz]/[11–30 Hz]). For our detailed analysis on tegus, we detected the peak of each power spectrum of the state of maximum power in the 10–20 Hz band and the crossing frequency between the two normalized power spectra in order to extract the band power ratio that maximizes the cluster detection. Based on the means of these values, we defined S2R, which is the mean power of the 10–22 Hz band divided by the sum of the 4–10 Hz and the 22–28 Hz bands (S2R = [10–22 Hz]/[(4–10 Hz) + (22–28 Hz)]). This ratio was calculated for the 24-h baseline of each animal on the chosen whitened electrode. A threshold was defined as the mean plus one standard deviation (Fig 3E). Every part of the signal above that threshold was considered as S2. If the S2 episodes were separated by less than 2 s, they were merged, and the episodes lasting less than 2 s were removed. The autocorrelation of Figs 1E, 1G and 4C was computed as described in Shein-Idelson and colleagues.

### Physiological measurements

The heart rate was extracted from the ECG electrode previously filtered with a high-pass filter (Fc 10 Hz, order 10). A peak detection was performed (threshold 100 μV, min interval between peak 0.7 s). The instantaneous heart rate was then computed by measuring the interval between peaks. The muscle tone was extracted from differential EMGs filtered with a high-pass filter (Fc 10 Hz, order 10). The muscle tone is the absolute value of the Hilbert transform of the signal was filtered with a mean filter (windows 0.5 s). The eye movement density was calculated from the EOG channels. The signal was filtered with a low-pass filter (Fc 10 Hz, order 10). Each part of the signal above 30 μV was considered as an eye movement. The density of eye movements corresponds to the number of eye movements occurring per min per state. Fig 1F, representing the phase histogram of the eye movements of the bearded dragon, was obtained by detecting the δ and β periods. The δ/β ratio was evaluated. Each δ period was extracted when the ratio was higher than the average and β periods when the ratio was lower than the average. Each cycle of δ–β periods was normalized between 0 and 2 π radians. The distribution of the occurrence of the ocular movements was evaluated relatively to this cycle.

### Sharp wave extraction

For the tegus, the HShW extraction was performed on all channels that were not considered as being in the CSF. The HShW detection algorithm was adapted from the spike-detection algorithm described by Quiroga and colleagues [40]. The HShWs were detected without any filter applied to the data. The threshold used was 10 times the signal-to-noise ratio, and 50 ms before and after the peak of the HShW was used for the waveform averaging. The channels kept for the analysis (baseline, sleep deprivation, and pharmacology) were the channels with the cleanest mean waveforms. The HShW density was evaluated by dividing the number of HShWs during a state by the duration of that state.

### Statistics

All the statistics were performed using Matlab. Wilcoxon signed-rank tests were used for single comparisons between mean parameters per state (Figs 2B, 2C, 2D, 4I, 6A, 6B, 6E, 6F and 6G). For multiple conditions with balanced designs, an analysis of variance with two factors (ANOVA2) was used followed by post hoc analysis using Fisher’s least significant difference procedure (Figs 4F, 4H, 5D, 5E, 5F, 7A, 7B, 7D and 7F). Each three-hour period data presented in Fig 4H were calculated, for each animal, by averaging 3 consecutive values from the sequential data of Fig 4G. For unbalanced designs, Kruskal-Wallis tests were performed and followed by post hoc analysis using Fisher’s least significant difference procedure. For the ANOVA, the normality of the data was tested with a Lilliefors test. When data were not normal, a Gaussian normalization centered on 0 with a variability of 0.2 was applied before any statistical test. The homoscedasticity was verified when needed using a Bartlett’s test. A difference was considered significant if the *p*-value was lower than 0.05 (* for *p* < 0.05, ** for *p* < 0.001, *** for *p* < 0.0001). All data are expressed as means ± standard error of the mean.

## Supporting information

S1 Fig Automated scoring over 24 h in animal #1. The figure represents 4 d of automated scoring. From top to bottom: the absolute EMG value in red and the threshold (black line) used for detecting AW bouts; the maximal amplitude of eye movement for a 1-s window and the threshold used (in black) for detecting eye movements; the interval between eye movement and the threshold (in black) used to score QW and SB periods; the hypnogram obtained from the automated scoring with QW and SB; the final automated hypnogram including the three states; a manual hypnogram representing the position of the animal, outside or inside the shelter. AW, active wake; EMG, electromyogram; QW, quiet wake; SB, sleep behavior.
(PDF)

S2 Fig Electrode position from MRI and CT scan. Frontal, sagittal, and horizontal slices of presurgical MRI merged with electrode segmented from a CT scan obtained after surgery for each brain region in each animal. CT, computed tomography.
(PDF)

S3 Fig Electrode sorting. Representation of the mean power spectral density between 10 and 45 Hz for all electrodes of all animals. On the left are the electrodes that were identified from the MRI and CT scan as being in the CSF. In the middle are the electrodes that are in the brain, and at the right, the electrodes with an undetermined position (ND). The black line represents the average plus one standard deviation of the power spectral density from the electrodes located in CSF. All electrodes with a cross are considered as not being in the brain and therefore were not considered for further processing. CSF, cerebral spinal fluid; CT, computed tomography; ND, undetermined position.
(PDF)

S4 Fig Electrode choice for S2 calculation. Mean power spectrum during SB for all electrodes (in blue) for all animals. In black, the values kept for the interpolation (red). A ratio that characterized the quantity of oscillation in the 10–20 Hz band is calculated for each electrode. To do so, the percentage of increase of the mean power spectrum between 10–20 Hz is computed compared to the interpolated curve. The title for each axis contains the animal number, the region recorded (based on the MRI and CT scan), the electrode name, and the ratio obtained. All electrodes considered as in the CSF are labeled at the upper right corner of the axis by a cross, whereas a red circle represent the electrodes with the higher ratio, chosen for the analysis of S2. CSF, cerebral spinal fluid; CT, computed tomography.
(PDF)

S1 Table Efficiency of the automated scoring. The table presents the mean and individual efficiency of the automatic scoring. The correct rate, the sensitivity, and the ability to score sleep correctly are presented. The SB epochs of the automated hypnogram are compared with the epochs’ scored “shelter” of the manual hypnogram. SB, sleep behavior.
(XLSX)

S1 Video Wake states. The video shows 5 min of the behavior and biological signals during the wake period of animal #1. It shows QW (in orange) and AW states (in blue). From the top to the bottom: the color hypnogram, the EMG filtered with a high pass at 10 Hz, the ECG filtered with a high pass at 10 Hz, the EOG filtered with low pass at 10 Hz, and the raw trace of an LFP recorded from the cMC and below its time frequency representation. The scales are in μV and Hz. The color scale is between −120 and −90 dB. The video was accelerated four times, and the time between two vertical lines is 2 s. AW, active wake; cMC, caudal medial cortex; ECG, electrocardiogram; EMG, electromyogram; EOG, electro-oculogram; LFP, local field potential; QW, quiet wake.
(AVI)

S2 Video Sleep states. The video represents 5 min of the behavior and biological signals during the sleep period of animal #1. It shows S1 (in green) and S2 (in red). From the top to the bottom: the color hypnogram, the EMG filtered with a high pass at 10 Hz, the ECG filtered with a high pass at 10 Hz, the EOG filtered with low pass at 10 Hz, and the raw trace of an LFP recorded from the cMC and below its time frequency representation. The scales are in μV and Hz. The color scale is between −120 and −90 dB. The video was accelerated four times, and the time between two vertical lines is 2 s. cMC, caudal medial cortex; ECG, electrocardiogram; EMG, electromyogram; EOG, electro-oculogram; LFP, local field potential; S1, sleep state 1; S2, sleep state 2.
(AVI)

S1 Data Quantitative data for each figure. (XLSX)

## Abbreviations

AW: active wake
CA1: cornu ammonis area 1
CA3: cornu ammonis area 3
cMC: caudal medial cortex
CSF: cerebral spinal fluid
CT: computed tomography
DVR: dorsoventricular ridge
ECG: electrocardiogram
EEG: electroencephalogram
EMG: electromyogram
EOG: electro-oculogram
FOV: field of view
HR-MRI: high-spatial–resolution magnetic resonance imaging
HShW: high-amplitude sharp wave
hSWP-R: hippocampal sharp-wave ripple
LFP: local field potential
MPSD: mean power spectrum density
NS: nucleus sphericus
PSD: power spectrum density
QW: quiet wake
REM: rapid eye movement
rMC: rorsal medial cortex
S1: sleep state 1
S2: sleep state 2
S2R: S2 detection ratio
SB: sleep behavior
SWS: slow-wave sleep
