# Supplementary figures and images for: Partial homologies between sleep states in lizards, mammals, and birds suggest a complex evolution of sleep states in amniotes
Source: PLoS Biol. 2018 Oct 11;16(10):e2005982. doi: 10.1371/journal.pbio.2005982 (PMC6181266; doi:10.1371/journal.pbio.2005982)

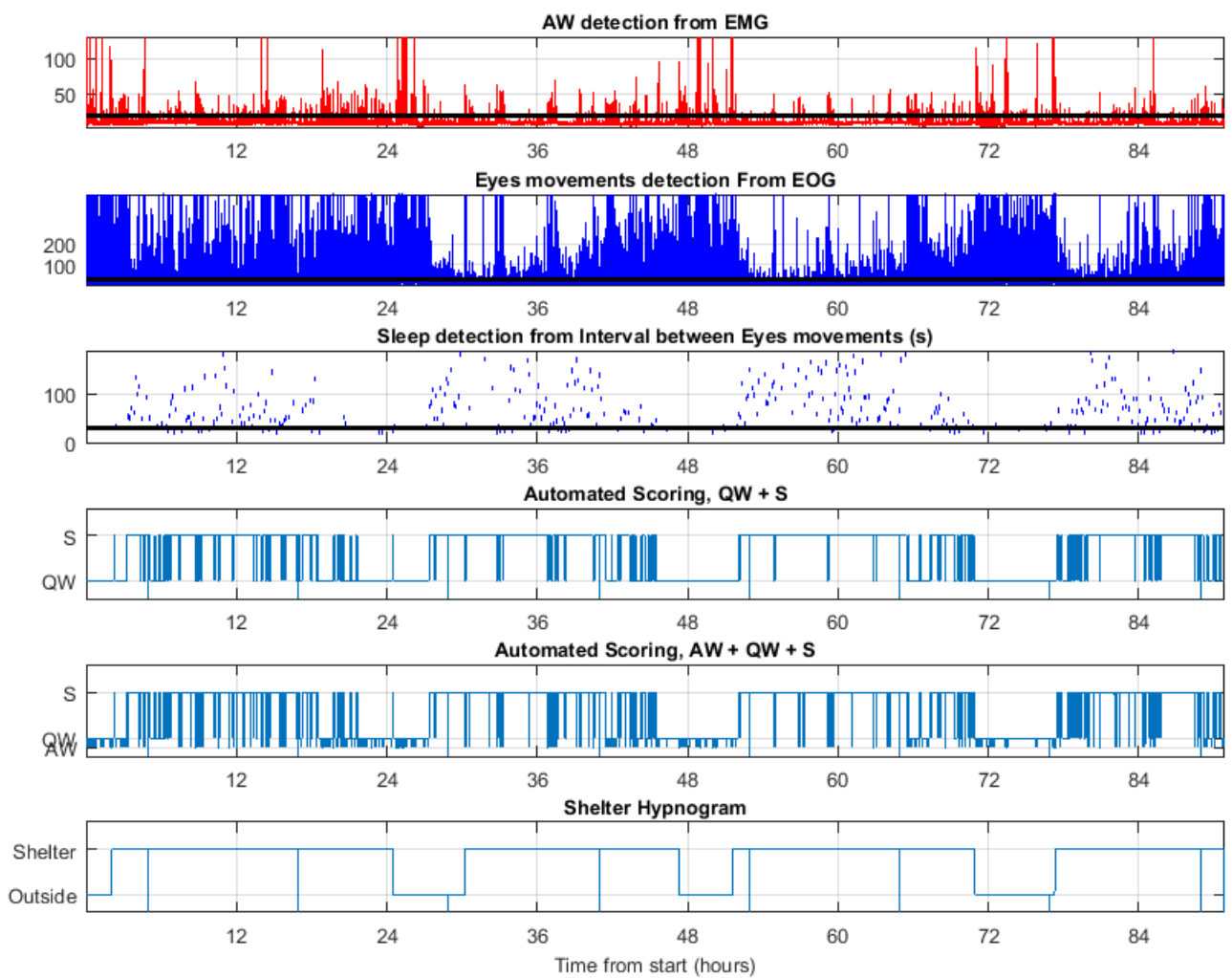

Supplement: S1 Fig — The figure represents 4 d of automated scoring. From top to bottom: the absolute EMG value in red and the threshold (black line) used for detecting AW bouts; the maximal amplitude of eye movement for a 1-s window and the threshold used (in black) for detecting eye movements; the interval between eye movement and the threshold (in black) used to score QW and SB periods; the hypnogram obtained from the automated scoring with QW and SB; the final automated hypnogram including the three states; a manual hypnogram representing the position of the animal, outside or inside the shelter. AW, active wake; EMG, electromyogram; QW, quiet wake; SB, sleep behavior. (PDF) [file pbio.2005982.s001.pdf]

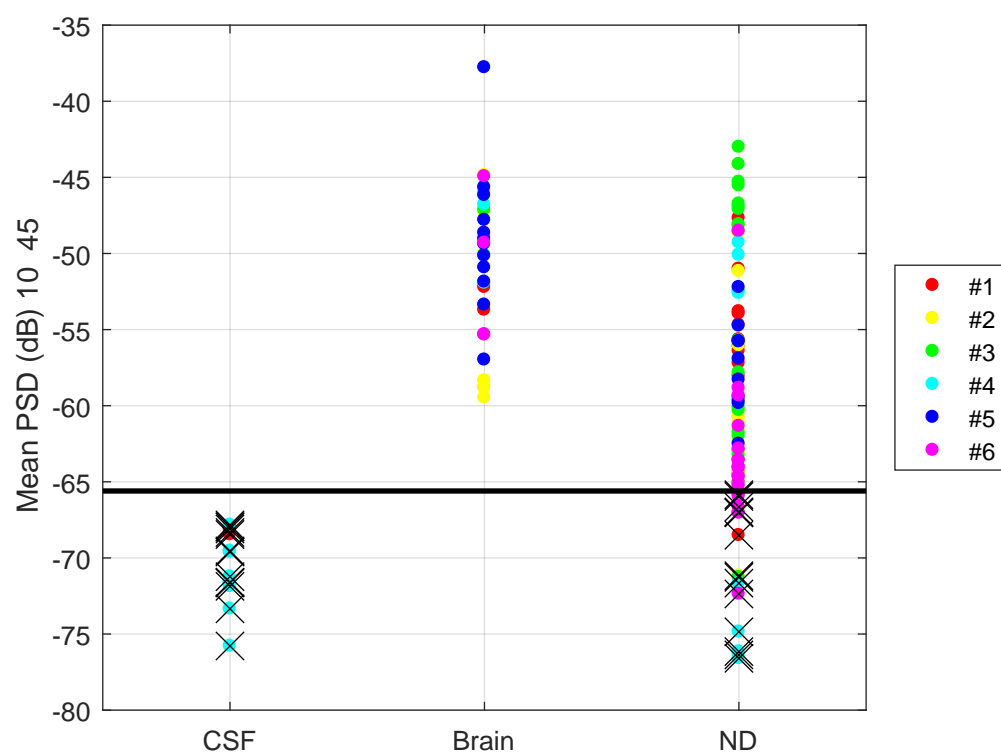

Supplement: S3 Fig — Representation of the mean power spectral density between 10 and 45 Hz for all electrodes of all animals. On the left are the electrodes that were identified from the MRI and CT scan as being in the CSF. In the middle are the electrodes that are in the brain, and at the right, the electrodes with an undetermined position (ND). The black line represents the average plus one standard deviation of the power spectral density from the electrodes located in CSF. All electrodes with a cross are considered as not being in the brain and therefore were not considered for further processing. CSF, cerebral spinal fluid; CT, computed tomography; ND, undetermined position. (PDF) [file pbio.2005982.s003.pdf]

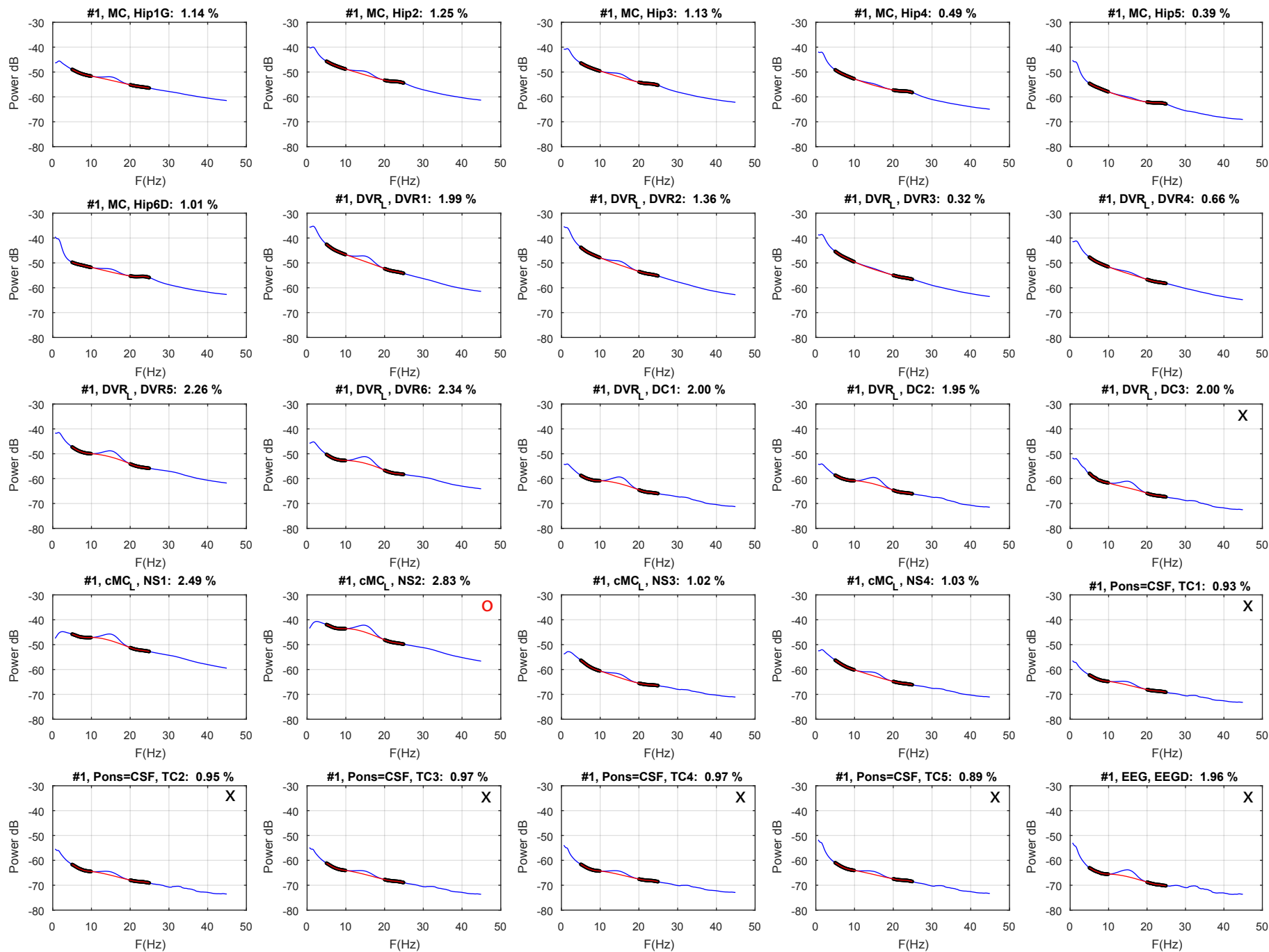

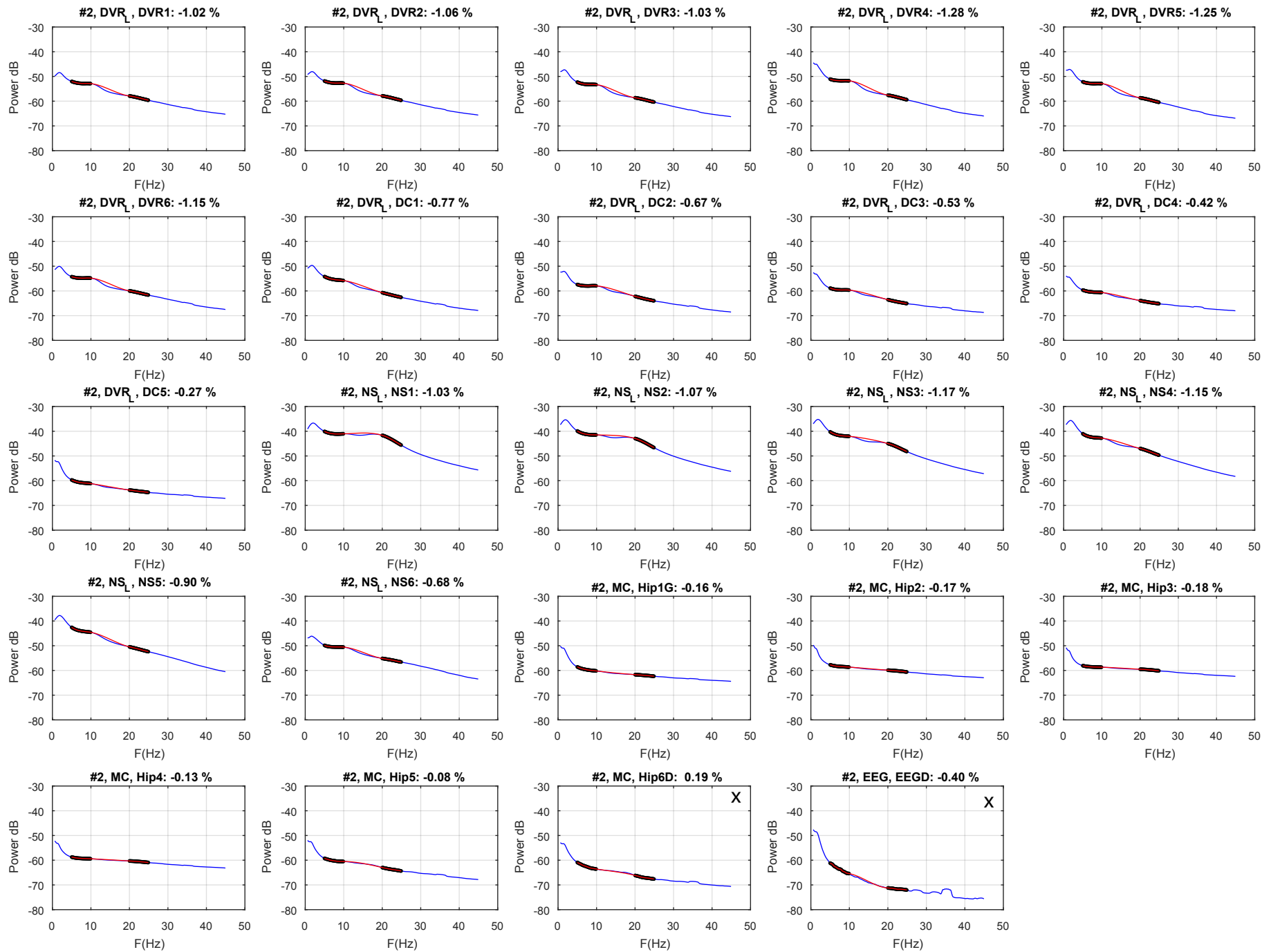

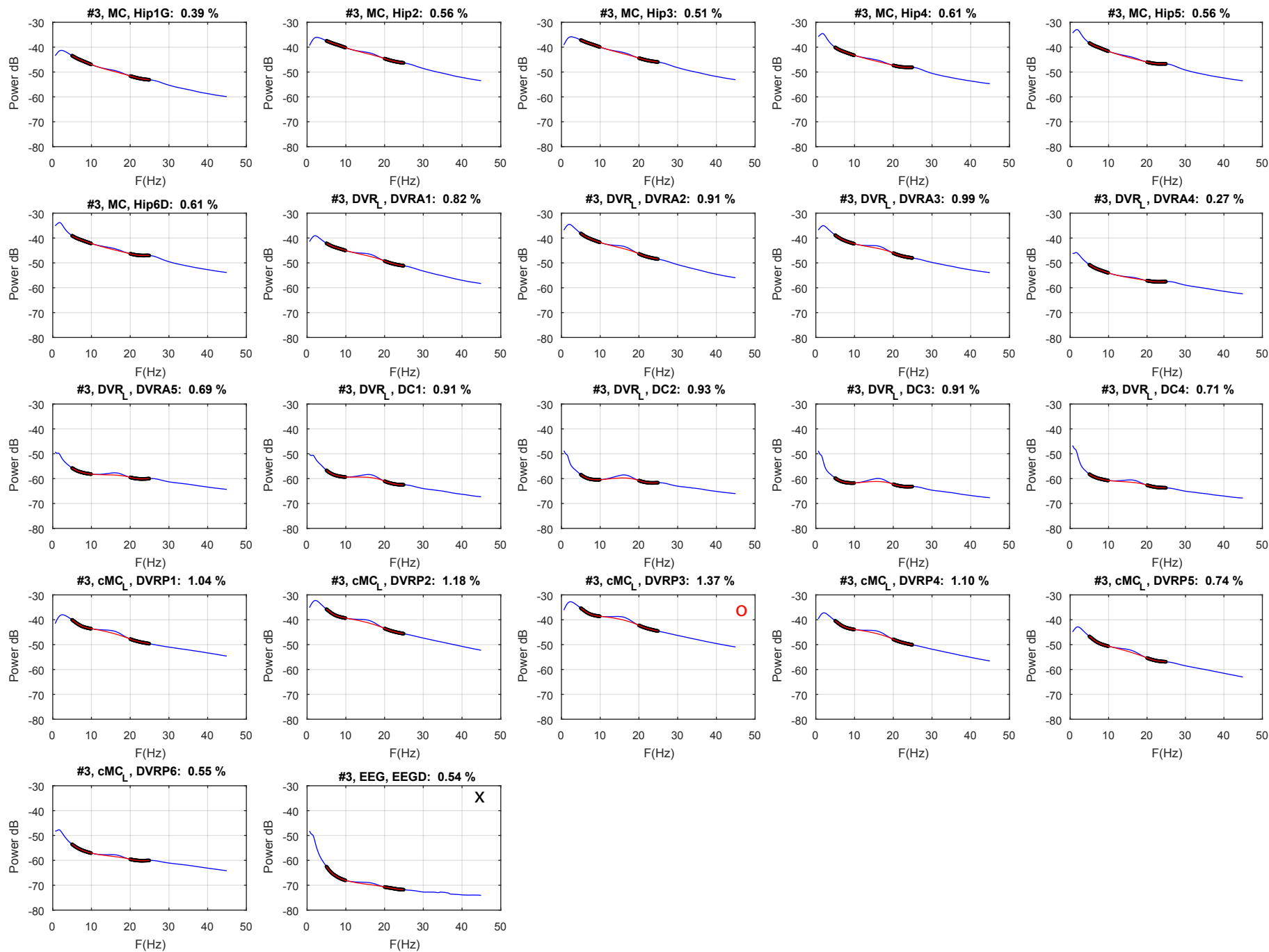

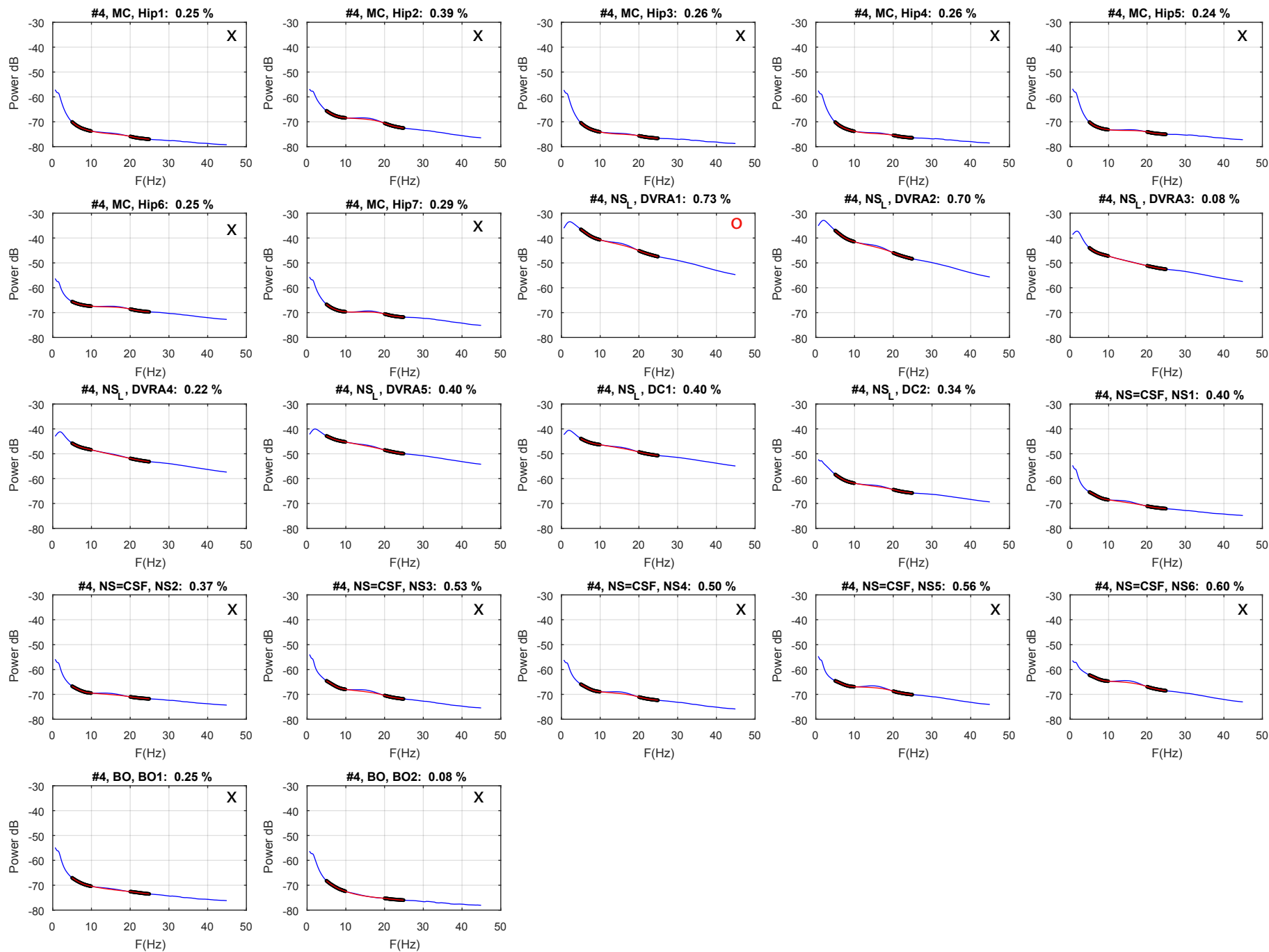

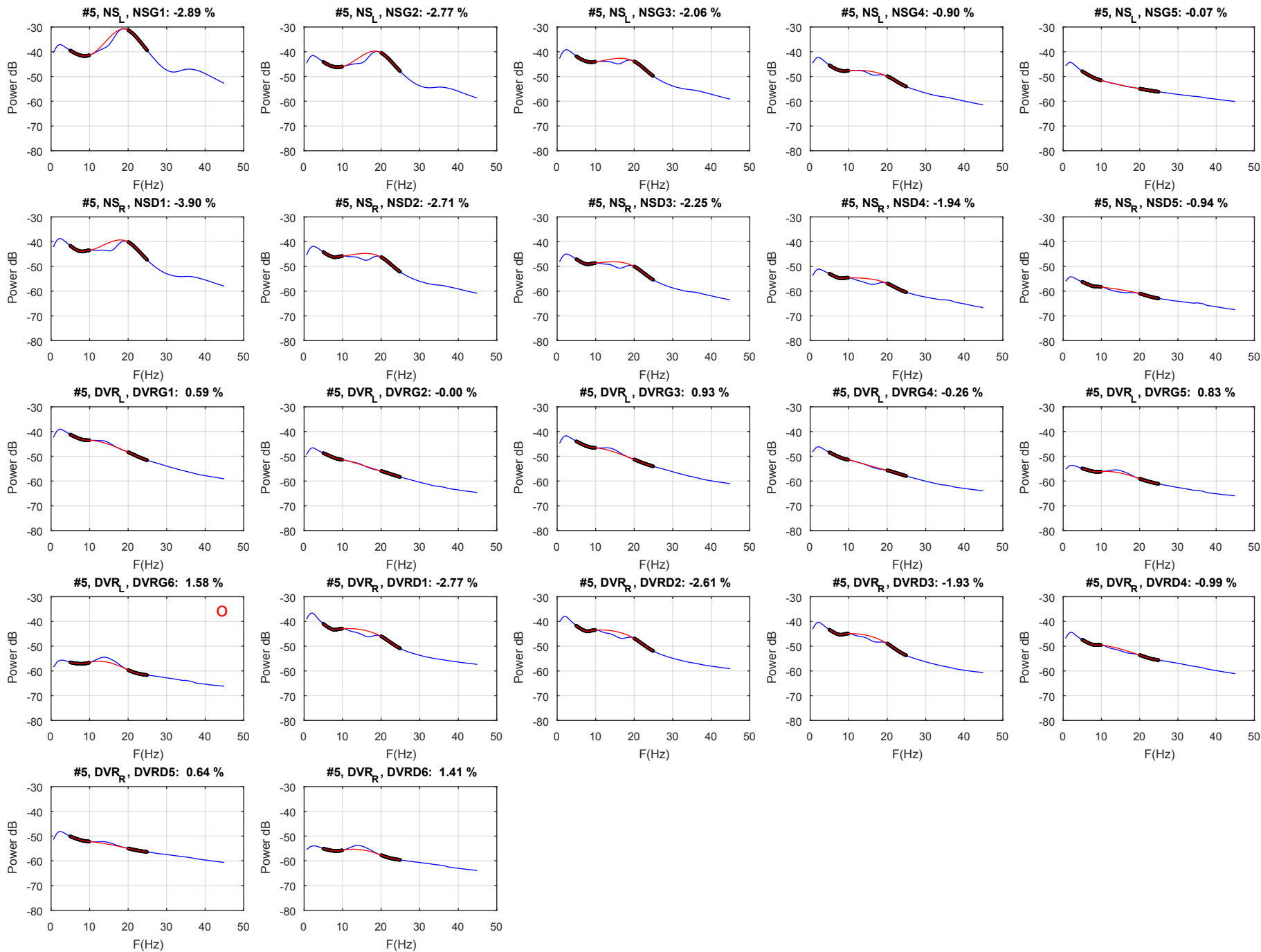

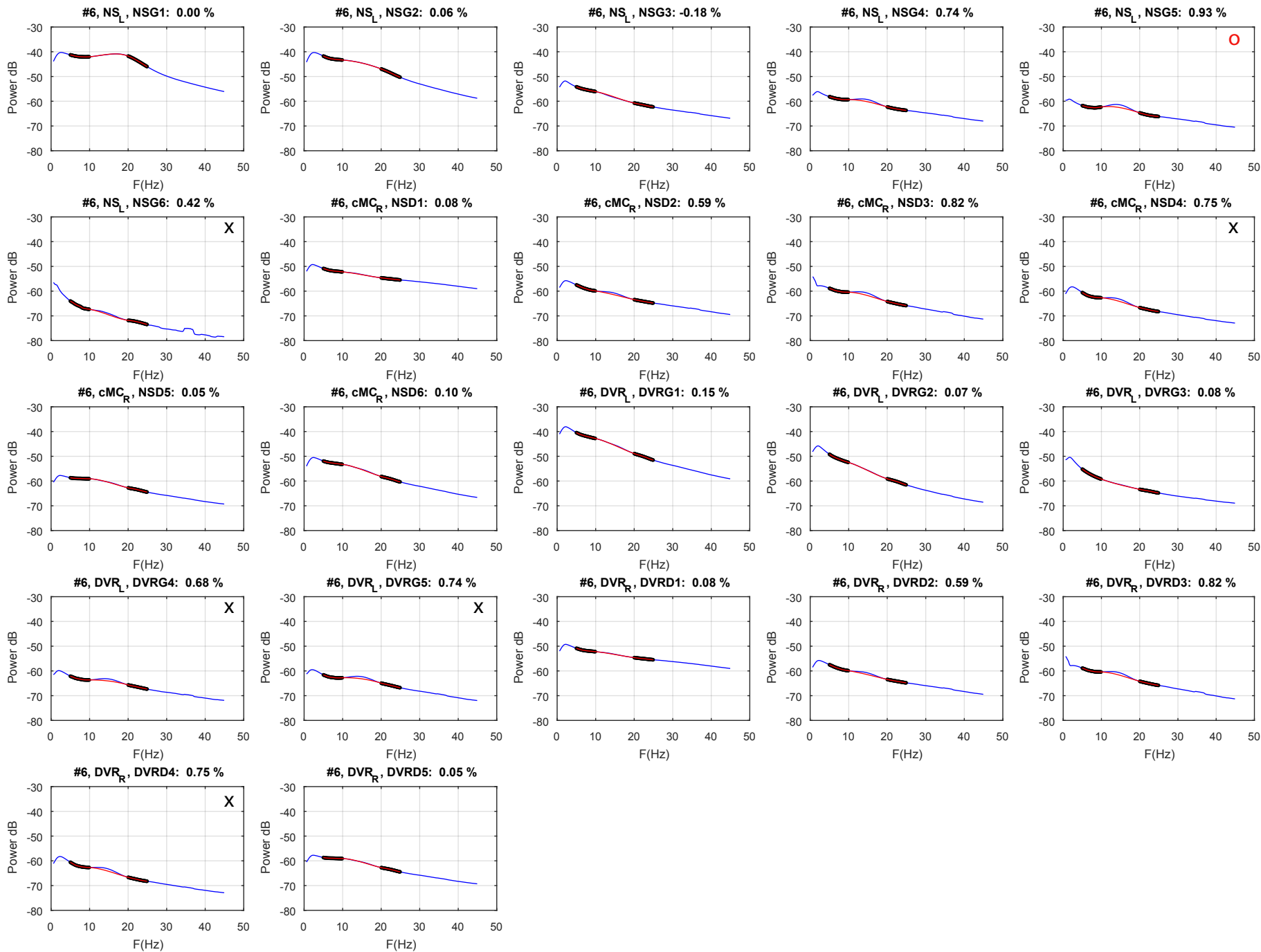

Supplement: S4 Fig — Mean power spectrum during SB for all electrodes (in blue) for all animals. In black, the values kept for the interpolation (red). A ratio that characterized the quantity of oscillation in the 10–20 Hz band is calculated for each electrode. To do so, the percentage of increase of the mean power spectrum between 10–20 Hz is computed compared to the interpolated curve. The title for each axis contains the animal number, the region recorded (based on the MRI and CT scan), the electrode name, and the ratio obtained. All electrodes considered as in the CSF are labeled at the upper right corner of the axis by a cross, whereas a red circle represent the electrodes with the higher ratio, chosen for the analysis of S2. CSF, cerebral spinal fluid; CT, computed tomography. (PDF) [file pbio.2005982.s004.pdf]
