# Supplementary material for: Partial homologies between sleep states in lizards, mammals, and birds suggest a complex evolution of sleep states in amniotes
Source: PLoS Biol. 2018 Oct 11;16(10):e2005982. doi: 10.1371/journal.pbio.2005982 (PMC6181266; doi:10.1371/journal.pbio.2005982)

Dorso Ventricular Ridge + Dorsal Cortex

#1

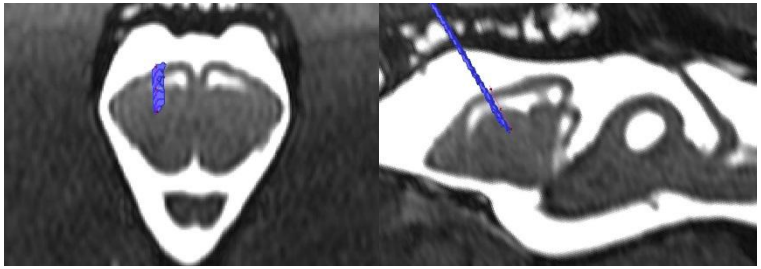

#2

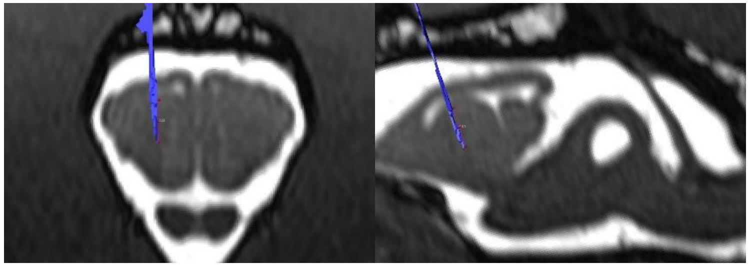

#3

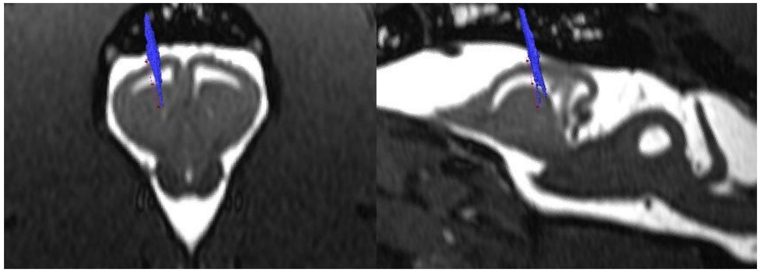

#5

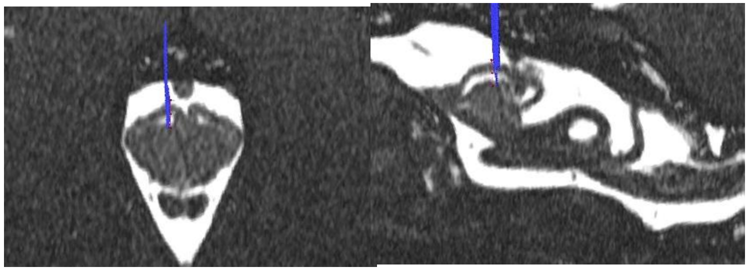

#5

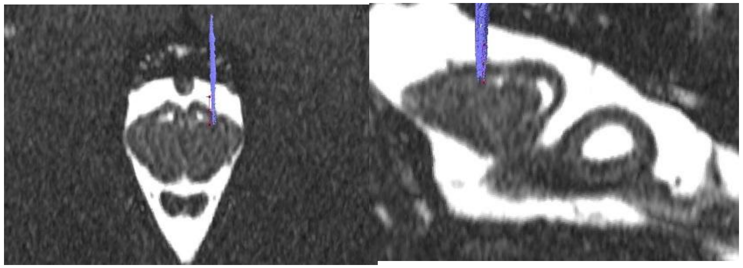

#6

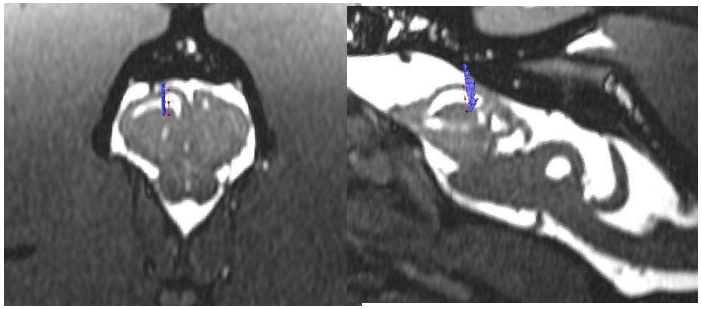

#6

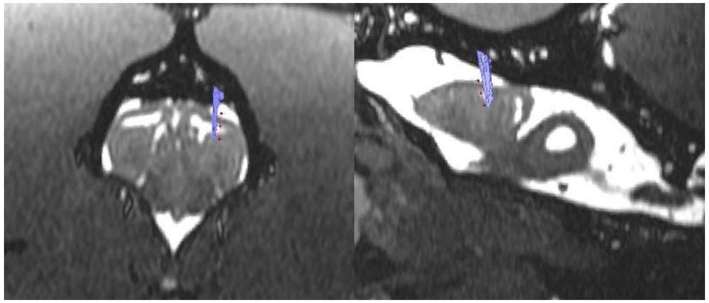

Medial Cortex

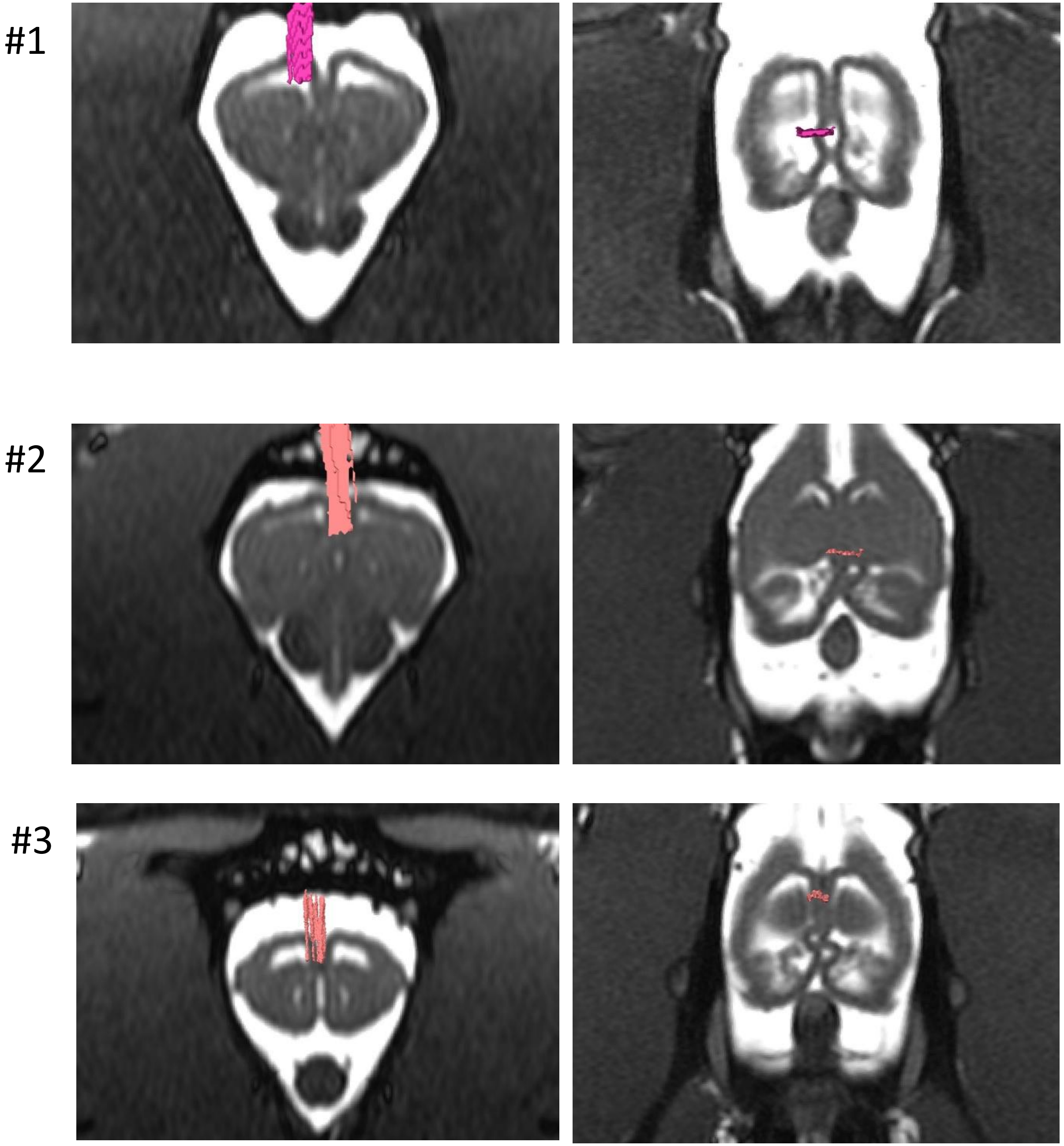

Nucleus sphericus without Dorsal cortex

#1

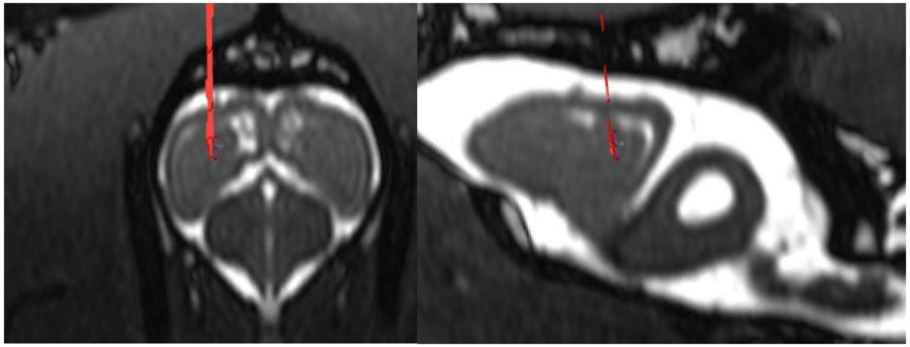

#4

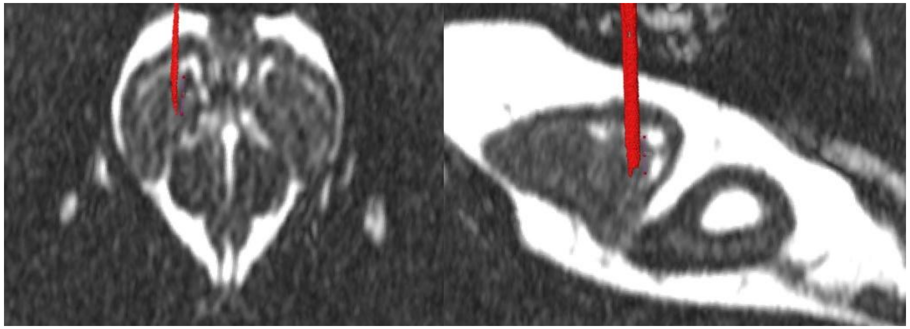

#4

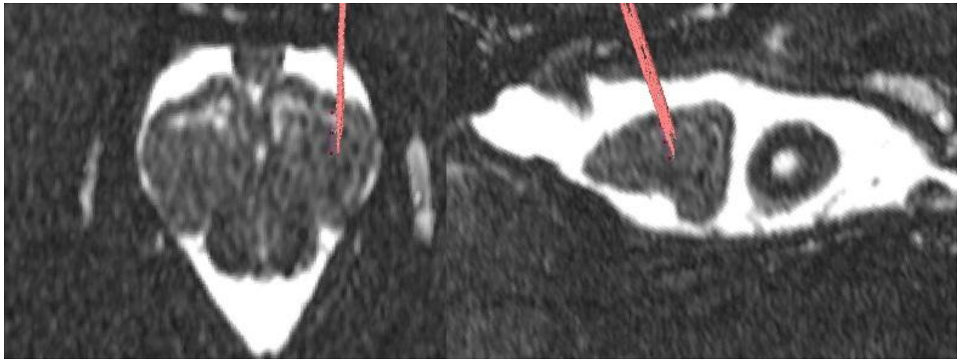

Nucleus sphericus with Dorsal cortex

#4

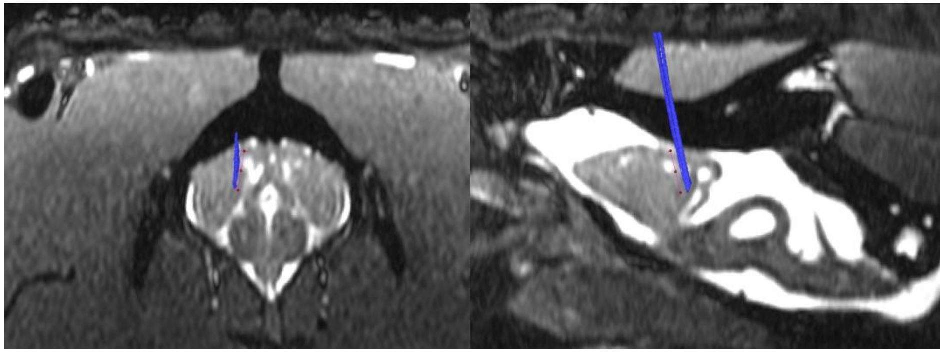

#6

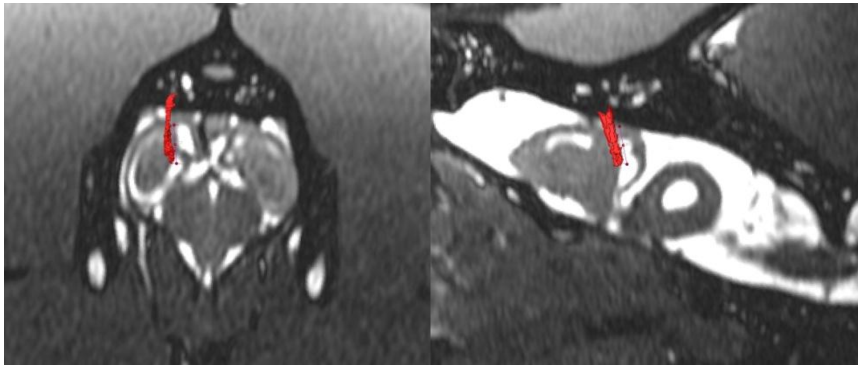

Caudal Medial Cortex

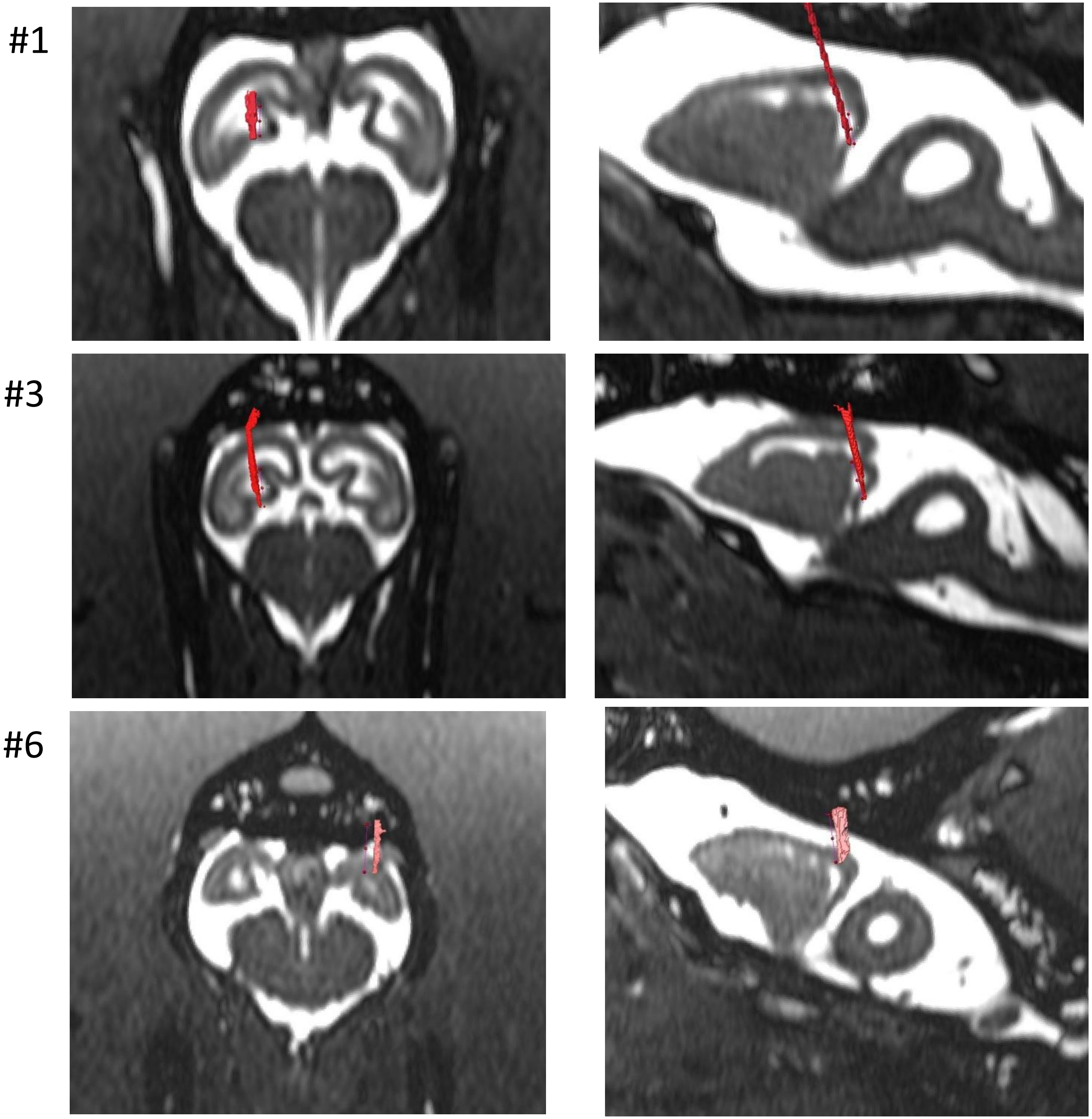

Supplement: S2 Fig — Frontal, sagittal, and horizontal slices of presurgical MRI merged with electrode segmented from a CT scan obtained after surgery for each brain region in each animal. CT, computed tomography. (PDF) [file pbio.2005982.s002.pdf]
